# Quantitative Three‐Dimensional Characterization of Facial Dysmorphism in Self‐Identified African‐American Individuals With Prenatal Alcohol Exposure

**DOI:** 10.1111/acer.70396

**Published:** 2026-08-07

**Authors:** Qing Li, Zahra Mozaffarian, Hanie Moghaddasi, Claire D. Coles, Julie A. Kable, Sarah N. Mattson, Jeffrey R. Wozniak, Kenneth Lyons Jones, Miguel del Campo, Leah Wetherill, Michael Suttie, the CIFASD Consortium

**Affiliations:** ^1^ Department of Orthopedic Surgery, State Key Laboratory of Complex Severe and Rare Diseases, Peking Union Medical College Hospital Chinese Academy of Medical Sciences & Peking Union Medical College Beijing China; ^2^ Nuffield Department of Women's & Reproductive Health University of Oxford Oxford UK; ^3^ Big Data Institute University of Oxford Oxford UK; ^4^ CAMS Oxford Institute, Chinese Academy of Medical Sciences & Peking Union Medical College University of Oxford Oxford UK; ^5^ Department of Psychiatry and Behavioral Sciences Emory University School of Medicine Atlanta Georgia USA; ^6^ Center for Behavioral Teratology, Department of Psychology San Diego State University San Diego California USA; ^7^ Department of Psychiatry & Behavioral Sciences University of Minnesota Minneapolis Minnesota USA; ^8^ Division of Dysmorphology and Teratology, Department of Pediatrics University of California San Diego (UCSD) La Jolla California USA; ^9^ Division of Genetics Rady Children's Hospital San Diego San Diego California USA; ^10^ Department of Medical and Molecular Genetics Indiana University School of Medicine Indianapolis Indiana USA

**Keywords:** 3D facial analysis, facial dysmorphism, fetal alcohol spectrum disorders, prenatal alcohol exposure

## Abstract

**Background:**

The objective of this study was to characterize facial dysmorphology in a self‐identified African‐American cohort of children with prenatal alcohol exposure (PAE) and to evaluate the utility of computer vision‐based methods for quantitative assessment and screening of facial form.

**Methods:**

A total of 307 self‐identified African‐American individuals were evaluated by expert dysmorphologists and classified according to PAE status as unexposed controls (controls), individuals with prenatal alcohol exposed without fetal alcohol syndrome (FAS)‐associated physical features (AE), or individuals with prenatal alcohol exposed with FAS‐associated physical features (AE‐FAS). Three‐dimensional (3D) facial images were analyzed using volumetric and measurement‐based approaches. Statistical shape modeling was applied to support surface‐based analysis, cluster identification, and group discrimination testing.

**Results:**

(i) Facial growth in the AE‐FAS group was significantly reduced. (ii) We observed a subgroup of AE clinically without FAS‐associated physical features who displayed an affinity to the AE‐FAS group in the unscaled models. (iii) Sex‐stratified analyses revealed alcohol‐associated alterations in sexual dimorphism of facial morphology, with a substantially stronger association between PAE and reduced facial growth in females than in males. (iv) Unique patterns of facial dysmorphology were exhibited in the AE group, showing underdevelopment of the zygomatic arch. (v) Using closest mean, support vector machine, and linear discriminant analysis, discrimination between AE‐FAS and controls was moderate.

**Conclusions:**

3D facial analysis facilitates recognition of potential sex‐related differences in facial morphology associated with PAE in African‐American children, a population underrepresented in the clinical literature. The distinct patterns of dysmorphology observed may contribute to improved phenotypic characterization and diagnostic support.

## Graphical abstract

Three‐dimensional facial analysis identified distinct patterns of facial dysmorphology associated with prenatal alcohol exposure in self‐identified African‐American children. Reduced facial growth was most evident in children with fetal alcohol syndrome‐associated physical features, and characteristic alterations were observed in the eyes, nose, and midface. These findings improve phenotypic characterization of prenatal alcohol exposure in an underrepresented population.

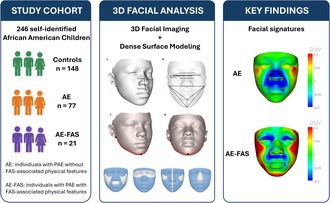

**Abbreviations**
3D: three‐dimensional
AE: prenatal alcohol exposed without FAS‐associated physical features
AE‐FAS: prenatal alcohol exposed with FAS‐associated physical features
ANCOVA: analysis of covariance
AUC: area under the receiver operating characteristic curve
BMI: body mass index
CI: canthal index
CIFASD: Collaborative Initiative on Fetal Alcohol Spectrum Disorders
CM: closest mean
DSM: dense surface model
FAS: Fetal alcohol syndrome
FASD: Fetal alcohol spectrum disorder
FDR: false discovery rate
FSW: facial signature weight
ICD: inner canthal distance
LDA: linear discriminant analysis
LFH: lower facial height
LPFL: left palpebral fissure length
MAL: mandibular arc length
MSW: mean signature weight
MV: mandibular volume
NH: nasal height
NHR: nasal height ratio
NL: nasal length
NP: nasal protrusion
OCD: outer canthal distance
PAE: prenatal alcohol exposure
PC: principal component
PCA: principal component analysis
PFL: palpebral fissure length
PL: philtrum length
ROC: receiver operating characteristic curve
RPFL: right palpebral fissure length
Shh: Sonic hedgehog
SVM: support vector machine
TFH: total facial height

## 1 Introduction

Prenatal alcohol exposure (PAE) leads to a continuum of structural, cognitive, and behavioral impairments collectively known as fetal alcohol spectrum disorders (FASD). Core diagnostic features include prenatal and postnatal growth restriction, distinctive craniofacial anomalies, and neurobehavioral deficits (Hoyme et al. 2016). Among these, shortened palpebral fissure length (PFL), a smooth philtrum, and a thin upper vermilion border are recognized as the cardinal facial markers of fetal alcohol syndrome (FAS) (Jones and Smith 1973).

Recent meta‐analyses estimate the global prevalence of FASD at approximately 7.7 per 1000, with rates rising 10‐ to 40‐fold in certain high‐risk or socioeconomically disadvantaged settings (Lange et al. 2017; Popova et al. 2019). Despite these prevalence rates, FASD remains substantially underdiagnosed, particularly in marginalized populations where access to specialized FASD assessment is limited and existing diagnostic tools may not be population‐appropriate (Oh et al. 2023; Rockhold et al. 2024).

One major challenge in FASD diagnosis lies in the reliance on craniofacial norms developed predominantly from Caucasian populations. Facial morphology differs substantially across ancestries, and applying non‐population‐matched standards, for example, using Caucasian PFL norms for African‐American children, risks misclassification, either missing affected individuals or overdiagnosing unaffected children (Hoyme et al. 2015; Moore et al. 2007). Heterogeneity within alcohol‐exposed populations presents a second challenge: many children with confirmed exposure show subtle dysmorphology without meeting full FAS criteria, while others appear phenotypically unaffected despite significant exposure. These complexities highlight the need for objective, data‐driven approaches to characterizing facial phenotypes.

African‐American children are historically underrepresented in FASD research, particularly in studies focused on facial morphology. Although some multisite cohorts have included individuals of African ancestry, their representation has typically been minimal (the largest published subset includes only ~25 individuals) (Jones et al. 2010; Moore et al. 2007; Yang et al. 2012) and no study to date has comprehensively examined FASD‐related facial morphology within this population. A recent multisite analysis including participants from the same Collaborative Initiative on Fetal Alcohol Spectrum Disorders (CIFASD) cohort as the present study found no significant association between race/ethnicity and FAS diagnosis after controlling for PAE and socioeconomic status (Oh et al. 2023). A detailed characterization of FASD‐related facial morphology in African‐American children is, therefore, not only overdue but also necessary to improve diagnostic accuracy and determine whether facial phenotypes associated with PAE manifest differently across ancestral groups.

Dense Surface Modeling and signature‐graph analysis have emerged as powerful tools for quantifying subtle, distributed facial shape differences from 3D images (Suttie et al. 2013, 2017, 2018). In our prior work on a mixed‐ancestry South African cohort and European ancestry children, these methods successfully identified characteristic signatures of FAS, discriminated FAS‐like and non‐FAS‐like subgroups within the alcohol‐exposed cohort, and showed high agreement with clinical diagnoses (Suttie et al. 2013).

Building on our previous studies on South African populations (Suttie et al. 2013), this study focuses on the distinct and understudied population of African‐American children. The primary goal of this study was to provide a comprehensive, data‐driven characterization of facial morphology associated with PAE in African‐American children using Dense Surface Modeling and signature‐graph analysis. Specifically, we aim to (1) evaluate whether previously identified FASD‐related facial dysmorphology patterns can be replicated in this population; (2) characterize facial morphology associated with PAE in African‐American children, including facial features beyond established diagnostic criteria; and (3) assess the extent to which facial morphology alone can capture homogeneity within the alcohol‐exposed cohort. By addressing these aims, this study seeks to advance ancestry‐appropriate, objective facial phenotyping approaches for FASD, improve diagnostic accuracy in historically underrepresented populations, and reduce disparities arising from the use of non‐population‐matched diagnostic standards.

## 2 Materials and Methods

### 2.1 Participants

Data were obtained from 307 participants recruited across four CIFASD study sites: Atlanta (*n* = 220), Minnesota (*n* = 40), San Diego (*n* = 38), and Los Angeles (*n* = 9). These data were collected as part of the ongoing CIFASD, encompassing phases 2 through 5. Participants were initially selected based on parent‐reported race, with children identified as Black or African‐American; 24 participants additionally reported mixed‐race ancestry.

Of the 307 participants available in the original dataset, 61 were excluded because they had incomplete information, a diagnosed genetic syndrome, or unknown prenatal alcohol exposure, preventing reliable assignment to one of the three study groups. The final analytical sample consisted of 246 participants, including 77 prenatal alcohol‐exposed participants without FAS‐associated physical features (AE), 21 prenatal alcohol‐exposed participants with FAS‐associated physical features (AE‐FAS), and 148 participants with minimal or no reported PAE (control). FAS‐associated physical features were determined according to the CIFASD dysmorphology criteria (Table S1), which include at least two of three cardinal facial features identified from physical dysmorphology exams (shortened PFL, thin upper lip vermillion, and smooth philtrum). Participants had a mean (standard deviation, SD) age of 11.6 (3.4) years (range, 5–18 years). No known sibling pairs were included in the analytical dataset, and all participants were treated as independent observations. The overall design and procedures of the CIFASD clinical studies have been described in detail elsewhere (Jones et al. 2006; Mattson et al. 2011).

### 2.2 Clinical Assessment

Participants underwent clinical evaluation for growth abnormalities and physical features associated with FASD by experienced dysmorphologists. Facial characteristics relevant to FAS diagnosis were assessed using established protocols: philtrum smoothness and upper lip vermilion thickness were rated with the lip–philtrum guide (Hoyme et al. 2015), while PFL was measured manually in millimeters using a ruler.

Individuals were classified as having either no/minimal PAE or having heavy PAE. Information regarding PAE was obtained through structured caregiver interviews and supplemented by review of medical, legal, and social service records at each site (Mattson et al. 2010). Heavy exposure was defined as 14 or more standard alcoholic drinks per week or more than four drinks per occasion at least once every week during pregnancy. Controls had minimal or no PAE. Minimal exposure was defined as less than one drink per week on average, with a maximum of two drinks on any one occasion. All other exposed individuals were excluded. Neuropsychological functioning was assessed using standardized test batteries. Neuropsychological data were available for 215 participants included in the final analytical sample, comprising 134 controls, 65 AE participants, and 16 AE‐FAS participants. The Differential Ability Scales, Second Edition (DAS‐II) (Elliott 1990), along with the California Verbal Learning Test–Children's Version (CVLT‐C) (Delis et al. 1994), was administered as part of a broader cognitive assessment protocol. The DAS‐II comprises multiple subtests evaluating verbal, nonverbal, and spatial reasoning and yields a General Conceptual Ability score analogous to an intelligence quotient score. The CVLT‐C provides a detailed evaluation of verbal learning and memory performance.

### 2.3 Facial Imaging

**FIGURE 1:**
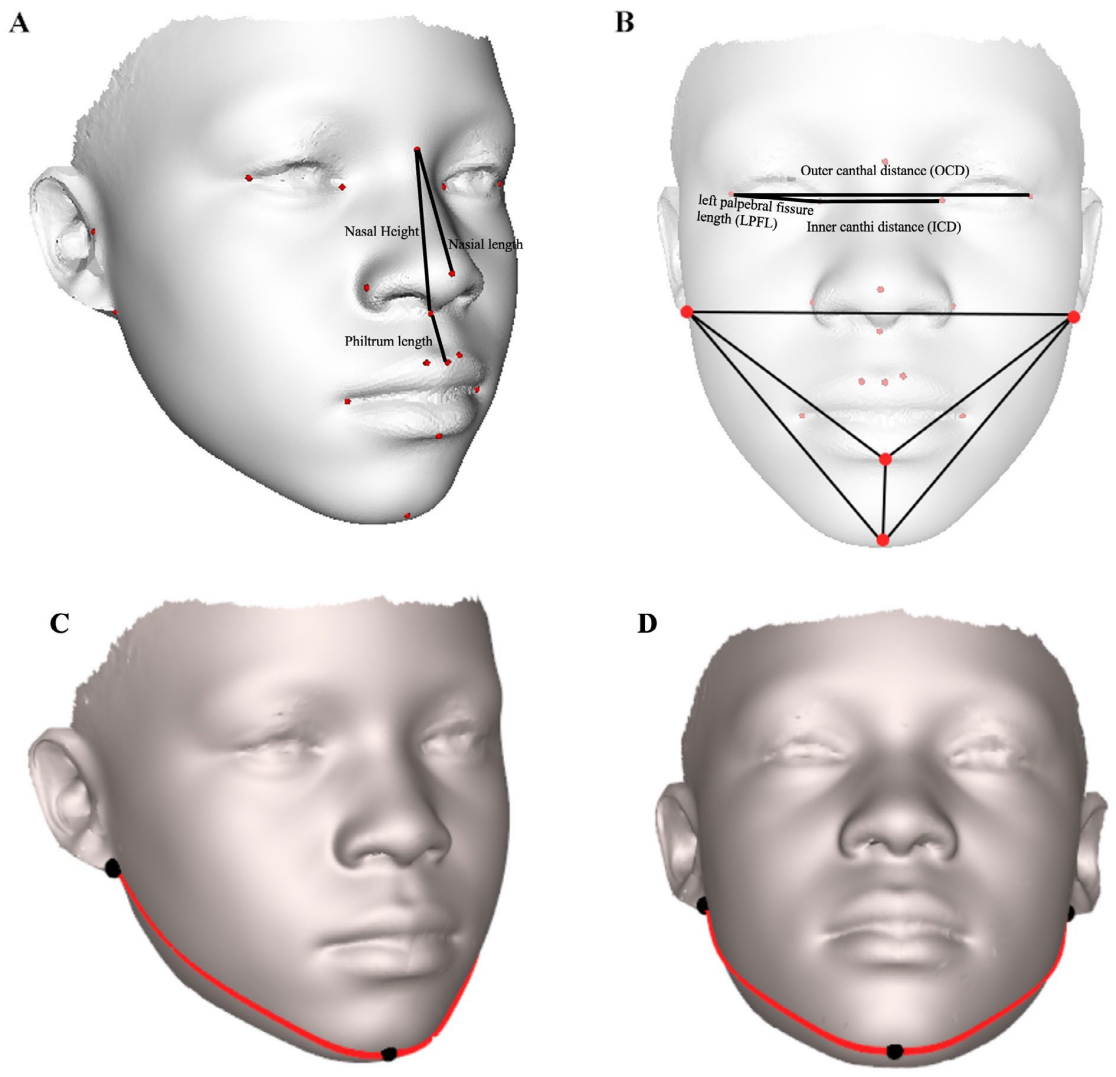
Visualization of facial landmarks and derived anthropometric measurements. (A) Locations of the 20 anatomical landmarks used for Dense Surface Modeling and anthropometric analysis, including bilateral (endocanthion, exocanthion, tragion, otobasion inferius, crista philtri, cheilion, alare) and midline landmarks (nasion, pronasale, subnasale, labiale superius, labiale inferius, gnathion). Key linear measurements are illustrated, including philtrum length, nasal height, and nasal length. (B) Ocular measurements, including inner canthal distance (ICD), outer canthal distance (OCD), and left palpebral fissure length (LPFL). Mandibular volume (MV) is estimated using a tetrahedral configuration defined by four landmarks: the bilateral otobasion inferius, labiale inferius (lower lip midpoint), and gnathion. (C, D) Illustration of mandibular arc length (MAL), defined as the surface contour distance along the inferior mandibular border between the bilateral otobasion inferius via the gnathion.

Three‐dimensional (3D) facial images were acquired using a commercial stereophotogrammetry system, a static tripod‐mounted system (3dMD; www.3dmd.com) capable of generating 180° ear‐to‐ear facial captures with submillimeter precision (geometric resolution < 0.2 mm). The resulting surface meshes comprised over 25,000 vertices. Facial surfaces were annotated using in‐house software (Facemark) by placing a standardized set of 20 anatomically defined landmarks (Figure 1). Landmarking was performed independently by two trained raters (M.S. and Q.L.) to ensure reliability. All landmark annotations underwent a final review by M.S. to ensure consistency and accuracy of landmark placement, and the quality of surface geometry was visually inspected.

### 2.4 Facial Measurements

Several linear and arc‐based facial measurements were derived from the annotated landmarks. The selection of these measurements was based on previous observations of facial dysmorphology across the FASD spectrum (Suttie et al. 2013, 2017, 2018), and on minor facial anomalies listed in the clinical diagnostic criteria for FAS (Hoyme et al. 2016). We implemented a novel 3D method for quantifying micrognathia (mandibular hypoplasia). This method computes the surface contour arc length from the left to the right lower ear attachment points through the gnathion (the most inferior point of the mandible), referred to as the mandibular arc, similar to that used in physical dysmorphology assessment (Hall et al. 1989; Olayemi 2011). A volumetric measurement of mandibular size was also calculated using an approach previously validated against CT images (Basart et al. 2018). Detailed definitions and computational procedures for all 15 facial measurements are provided in the Supporting Information Methods S1.

### 2.5 Dense Surface Modeling

**FIGURE 2:**
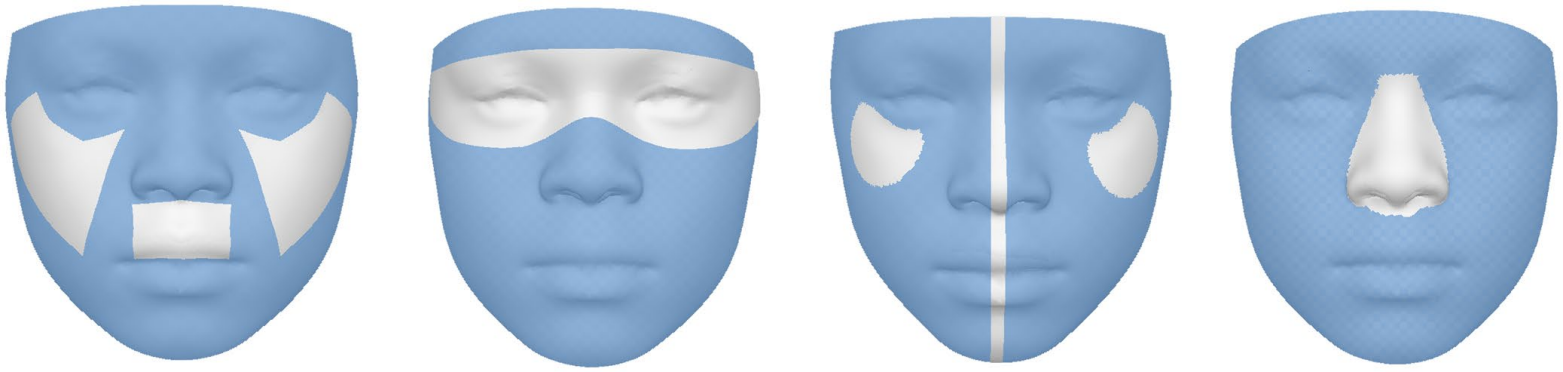
Visualization of facial regions used for DSM models. Individual anatomical facial regions used for DSM modeling, representing: Lip‐philtrum, malar region (left), eyes (middle‐left), thin‐profile, zygoma (middle‐right), and nose (right).

Dense Surface Models (DSMs) were generated for full face and selected facial regions (eyes, malar, nose, lip‐philtrum, profile, and zygomatic arch; Figure 2) using bespoke in‐house software (Hammond 2007; Hammond and Suttie 2012). This approach quantifies facial morphology by applying principal component analysis (PCA) to thousands of densely corresponded surface points derived from 3D facial scans. Prior to modeling, surfaces were aligned using thin‐plate spline warping based on a sparse set of 20 manually annotated anatomical landmarks.

Two types of models were constructed: combined shape‐and‐size DSMs to capture overall facial growth patterns, and shape‐only DSMs to isolate morphological variation independent of scale. In the shape‐and‐size models spanning a range of ages, overall facial size will typically account for the largest proportion of variance, with the first principal component (PC1) primarily reflecting growth‐related changes. For the shape‐only DSMs, landmark configurations were first aligned to a reference template using a Procrustes‐based similarity transformation, which was subsequently applied to the full 3D surfaces to remove size effects prior to PCA.

### 2.6 Facial Signature

Age‐matched reference faces were generated by computing running averages from sets of 35 control participants with contiguous ages. These average faces served as normative comparators against which subgroup mean faces were evaluated. For each analysis, point‐wise surface displacements were calculated by comparing each point on a subgroup mean face to the corresponding point on the age‐matched reference surface. These displacements were normalized and contrasted with the equivalent variability observed within the reference group, yielding quantitative facial signatures that capture deviations from typical facial morphology.

Facial signatures were visualized using color‐coded heatmaps, in which both hue and intensity indicate the magnitude and direction of surface displacement relative to the reference. Warmer colors (red) denote inward displacement corresponding to reduced feature prominence, cooler colors (blue) indicate outward displacement or feature enlargement, and green represents minimal or no deviation from the reference set.

To quantify facial dysmorphology, surface displacement metrics were computed for the entire face as well as for selected facial regions (eyes, malar, nose, lip–philtrum, profile, and zygomatic arch). Dysmorphism magnitude was summarized using the facial signature weight (FSW), defined as the square root of the summed squared normalized displacements across all surface points, providing a direction‐independent measure of overall deviation. The mean signature weight (MSW) was calculated as the average normalized displacement across points, capturing both the direction and magnitude of shape change. These measures were used to construct a connected facial signature graph in which each facial signature was linked to its nearest neighbor, forming clusters of phenotypically similar faces (Hammond and Suttie 2012). The resulting graphs were visualized using Graphviz (https://graphviz.org). Following methodologies from previous studies of alcohol‐exposed populations (Suttie et al. 2013, 2018), we characterized facial homogeneity within the AE group by examining graph structure and node connectivity. Individual affinity to AE‐FAS was assessed by subdividing the graph according to connectivity patterns. The largest connected component of AE individuals that did not include or connect to any AE‐FAS nodes was defined as the “non‐FAS‐like” division. In contrast, AE nodes located within or connected to components containing AE‐FAS nodes were classified as “FAS‐like,” reflecting their proximity to FAS‐associated facial phenotypes.

### 2.7 Statistical Analysis

All statistical analyses were conducted in the full sample and repeated in sex‐stratified analyses. Group differences were assessed using analysis of covariance (ANCOVA), with separate models fitted for each outcome measure. These analyses evaluated differences in facial growth captured by the PC1, additional leading principal components (PC2–PC3), FSW, MSW, and 15 landmark‐derived facial measurements across the three diagnostic groups.

For each outcome, the diagnostic group was included as the main effect, while age, sex, and body mass index (BMI) *z* scores were included as covariates to account for known influences on growth and facial morphology. BMI‐for‐age *z* scores were calculated according to the World Health Organization 2007 growth reference using the anthroplus R package. Overall group differences were first evaluated using ANCOVA. To account for multiple testing across facial morphology outcomes, false discovery rate (FDR) correction was applied across all scaled and unscaled principal‐component‐based measures (PC1–PC3) and landmark‐derived facial measurements. When significant overall group effects were observed, adjusted group means were estimated and post hoc pairwise comparisons between diagnostic groups were performed. Pairwise differences were reported as adjusted mean differences, and Bonferroni‐corrected *p* values were used to account for multiple pairwise comparisons within each outcome measure.

Because participants were recruited across multiple CIFASD sites, a sensitivity analysis was performed by repeating the primary ANCOVA models with recruitment site included as an additional covariate to assess the robustness of the findings. An additional sensitivity analysis was performed after excluding participants who reported mixed‐race ancestry.

For neurobehavioral analyses, group differences were evaluated using ANCOVA with adjustment for age, sex, and BMI *z* score. Given the larger number of neurobehavioral outcomes examined, FDR correction was applied across neurobehavioral variables. Formal group‐by‐sex interaction analyses were performed as supplementary analyses.

All statistical analyses were performed in R. ANCOVA models were fitted using base R, and estimated marginal means and post hoc pairwise comparisons were obtained using the emmeans package.

### 2.8 Face‐Based Classification Analyses

The agreement of clinical categorization and classification using DSM representations of facial shape was estimated using a multifold supervised learning framework. Classification performance was evaluated using 20 random stratified splits, with 90% of participants used for training and the remaining 10% retained as an unseen test set in each split. Stratified sampling preserved the relative proportions of the clinical groups across the training and test sets.

Three classification approaches were applied: closest mean classification, linear discriminant analysis (LDA), and support vector machines (SVM). Closest mean classification assigned individuals to the group whose mean facial representation was most similar. LDA identified linear combinations of DSM‐derived facial features that maximized between‐group differences relative to within‐group variation. SVM classification was used to identify separating boundaries between groups based on facial shape representations.

Classification analyses were performed using DSM‐derived facial PCs representing shape variance of the full face. Principal components were generated separately for each random train–test split and subsequently used for the discrimination analyses rather than being derived once from the complete dataset prior to splitting. Receiver operating characteristic (ROC) curve analysis was used to assess classification performance, which was summarized using the area under the ROC curve (AUC).

## 3 Results

### 3.1 Sample Characteristics

The final analytical sample comprised 148 unexposed controls (controls), 77 prenatal alcohol‐exposed participants (AE), and 21 prenatal alcohol‐exposed participants with FAS‐associated physical features (AE‐FAS) meeting CIFASD criteria (Table S1).

Detailed sample characteristics are presented in Table 1. There were no significant differences among the three groups in age or sex distribution (*p* > 0.05). Significant differences were observed for height, weight, and BMI *z*‐score. Height was lower in the AE‐FAS group compared with both the AE and control groups (*p* < 0.01). Weight was higher in controls than in the alcohol‐exposed groups (*p* < 0.001). BMI *z* scores were higher in controls than in the AE‐FAS group (*p* < 0.01). Corresponding boxplots are provided in the Figure S1.

**TABLE 1:** Sample characteristics.

	Control (*n* = 148)	AE (*n* = 77)	AE‐FAS (*n* = 21)	Test statistics
Age at 3D photo (years)	12.0 ± 3.5	11.4 ± 3.5	10.6 ± 3.2	*F*(2, 243) = 1.99
Sex (female/male, % female)	71/77 (48.0%)	37/40 (48.1%)	6/15 (28.6%)	*χ* ^2^(2) = 2.92
Height (cm)	150.9 ± 20.0	146.9 ± 19.8	134.1 ± 18.4	*F*(2, 243) = 6.91**
Weight (kg)	52.9 ± 25.9	45.3 ± 17.9	33.1 ± 15.6	*F*(2, 243) = 8.20***
BMI *z* score	0.9 ± 1.4	0.7 ± 1.3	−0.2 ± 1.3	*F*(2, 243) = 5.86**

*Note:* Values are mean ± standard deviation (SD) or *n* (%). *p < 0.05; **p < 0.01; ***p < 0.001.

### 3.2 Facial Measurements

A total of 20 anatomical landmarks were manually annotated on each 3D facial surface. From these landmarks, 15 linear, volumetric, and derived measurements were derived to characterize facial features commonly implicated in FASD (Figure 1). These measurements included left and right PFL (LPFL and RPFL), PFL, inner canthal distance (ICD), outer canthal distance (OCD), canthal index (CI), nasal length (NL), nasal height (NH), nasal protrusion (NP), philtrum length (PL), nasal height ratio (NHR), total facial height (TFH), lower facial height (LFH), mandibular volume (MV), and mandibular arc length (MAL).

Group differences in facial measurements were evaluated using ANCOVA across the three study groups, with age, sex, and BMI *z* scores included as covariates. Twelve of the 15 measurements showed significant overall group effects (Table 2). Post hoc analyses were subsequently performed to examine pairwise differences between groups, with detailed results reported in Table S1. Most significant differences reflected reduced facial dimensions in the AE‐FAS group relative to controls. Specifically, LPFL and RPFL, OCD, NL, TFH, LFH, MV, NP, and MAL were significantly smaller in the AE‐FAS group. The AE group generally showed intermediate values, often lying between controls and the AE‐FAS group, with smaller effect sizes. PL, CI, and NHR did not demonstrate significant overall group differences.

**TABLE 2:** ANCOVA results for unscaled and scaled facial measurements across control, AE, AE‐FAS groups.

	Control (*n* = 148)	AE (*n* = 77)	AE‐FAS (*n* = 21)	*F*(2, 243)
Unscaled measurements				
LPFL	28.1 ± 2.1	27.6 ± 1.7	26.3 ± 2.0	11.19***
RPFL	28.5 ± 2.1	28.0 ± 1.7	26.4 ± 1.9	16.61***
PFL	28.3 ± 2.0	27.8 ± 1.6	26.3 ± 1.8	15.76***
ICD	35.5 ± 3.4	34.7 ± 3.1	33.5 ± 2.6	5.77**
OCD	89.9 ± 6.0	88.4 ± 4.6	84.0 ± 5.2	21.80***
CI	0.39 ± 0.02	0.39 ± 0.02	0.4 ± 0.02	0.69
NH	49.3 ± 4.6	49.0 ± 4.7	46.1 ± 4.4	6.95**
NL	41.8 ± 4.5	41.2 ± 4.9	38.5 ± 4.1	8.25***
NP	12.4 ± 2.2	12.3 ± 2.2	10.3 ± 1.7	20.07***
PL	14.6 ± 3.0	15.4 ± 2.6	14.6 ± 3.1	2.51
NHR	0.45 ± 0.02	0.45 ± 0.02	0.45 ± 0.02	0.81
TFH	110.9 ± 9.8	109.8 ± 9.9	102.1 ± 8.8	19.12***
LFH	63.9 ± 6.9	63.7 ± 6.9	58.7 ± 5.3	13.16***
MV	69527.7 ± 18672.6	66766.6 ± 17525.4	55177.3 ± 11439.7	15.89***
MAL	254.6 ± 23.6	247.2 ± 20.8	233.2 ± 19.2	33.12***
PC1	0.21 ± 1.02	−0.04 ± 0.89	−0.87 ± 0.88	58.22***
PC2	0.08 ± 0.92	−0.14 ± 1.20	−0.07 ± 1.01	1.62
PC3	0.03 ± 1.03	−0.23 ± 1.07	0.18 ± 0.72	2.36
Scaled measurements				
LPFL	26.2 ± 1.5	26.3 ± 1.7	26.3 ± 1.9	0.09
RPFL	26.6 ± 1.5	26.7 ± 1.6	26.4 ± 1.8	0.31
PFL	26.4 ± 1.3	26.5 ± 1.6	26.3 ± 1.8	0.11
ICD	33.1 ± 2.4	33.0 ± 2.4	33.5 ± 1.8	0.45
OCD	83.9 ± 3.1	84.0 ± 3.6	84.1 ± 3.9	0.04
CI	0.39 ± 0.02	0.39 ± 0.02	0.4 ± 0.02	0.69
NH	46.0 ± 3.0	46.5 ± 3.4	46.1 ± 2.9	0.64
NL	38.9 ± 3.0	39.1 ± 3.6	38.4 ± 2.7	0.45
NP	11.5 ± 1.5	11.6 ± 1.6	10.2 ± 1.4	10.16***
PL	13.6 ± 2.5	14.6 ± 2.2	14.5 ± 2.8	4.98**
NHR	0.45 ± 0.02	0.45 ± 0.02	0.45 ± 0.02	0.81
TFH	103.3 ± 4.0	104.1 ± 5.4	102.0 ± 5.2	2.32
LFH	59.5 ± 3.6	60.4 ± 4.2	58.6 ± 2.7	2.98
MV	55172.4 ± 6402.2	55922.1 ± 7164.2	54532.0 ± 5785.8	0.58
MAL	237.1 ± 8.4	234.1 ± 9.2	232.9 ± 6.4	6.22**
PC1	0.05 ± 0.90	−0.10 ± 1.18	0.14 ± 1.13	0.94
PC2	−0.07 ± 1.03	−0.02 ± 0.89	0.55 ± 0.84	4.37**
PC3	0.18 ± 0.99	−0.26 ± 1.16	−0.28 ± 0.79	6.79**

*Note:* Unscaled measurements represent original facial dimensions incorporating both size and shape variation, whereas scaled measurements were normalized for overall facial size to capture shape‐only variation. Values are mean ± SD. *p < 0.05; **p < 0.01; ***P < 0.001.

Abbreviations: CI, canthal index; ICD, inner canthal distance; LFH, lower facial height; LPFL, left palpebral fissure length; MAL, mandibular arc length; MV, mandibular volume; NH, nasal height; NHR, nasal height ratio; NL, nasal length; NP, nasal protrusion; OCD, outer canthal distance; PC, principal component; PFL, palpebral fissure length (mean of left and right); PL, philtrum length; RPFL, right palpebral fissure length; TFH, total facial height.

To assess shape differences independent of overall facial size, size‐normalized (scaled) versions of the measurements were calculated after removing overall size effects. Following size normalization, only three measurements (PL, NP, and MAL) remained significantly different between groups (Table 2; Table S2; Figure S2).

To assess the robustness of the findings, sensitivity analyses were performed by (1) including recruitment site as an additional covariate and (2) excluding participants who reported mixed‐race ancestry. Neither analysis materially altered the results, and the overall pattern of significant findings remained unchanged (Tables S3 and S4).

### 3.3 Facial Growth and Dysmorphism Across the Fetal Alcohol Spectrum

**FIGURE 3:**
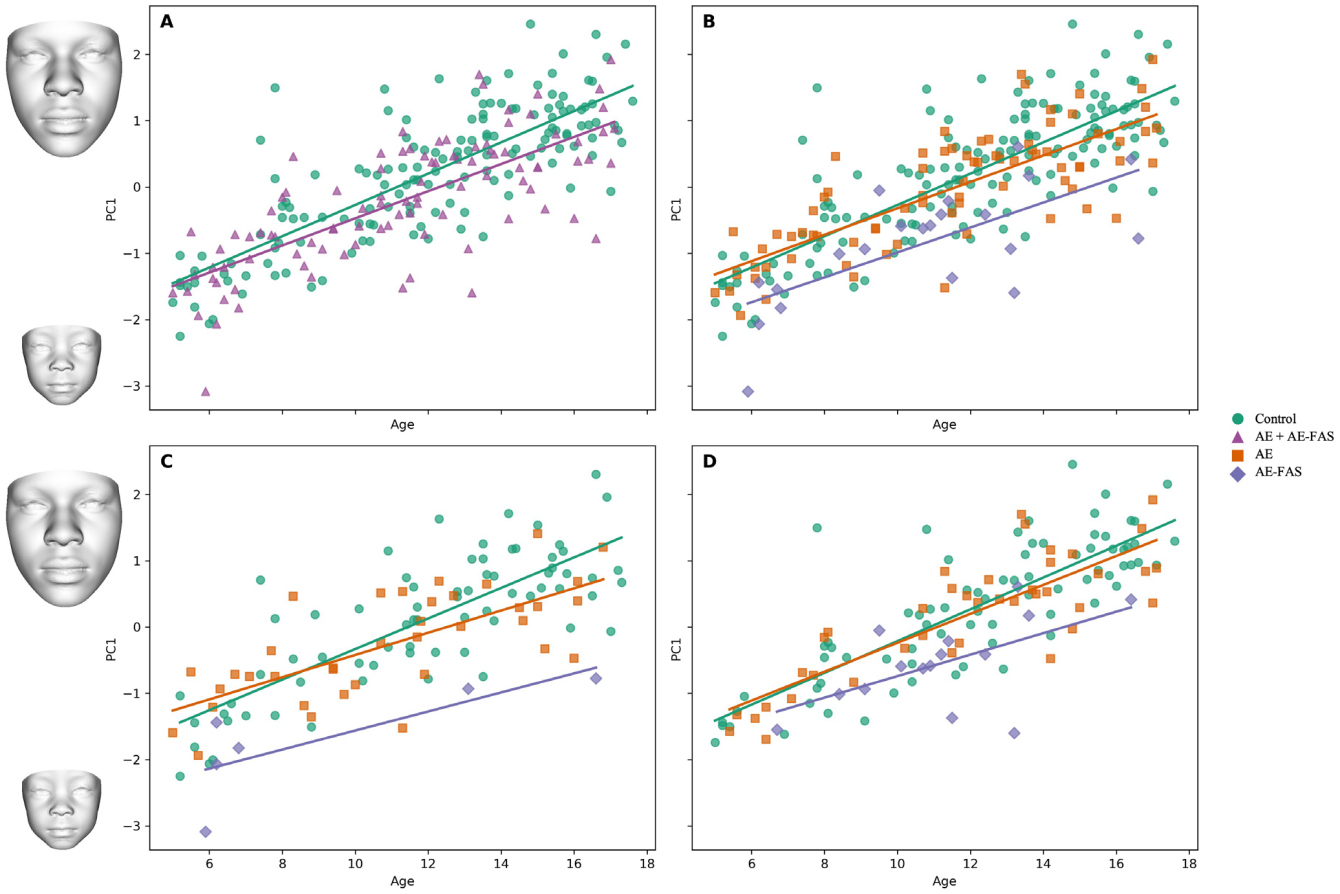
Comparison of facial growth across study groups. Facial growth was indexed by the first principal component (PC1) derived from the unscaled model. (A) Comparison of PC1 between controls and the combined alcohol‐exposed group (AE + AE‐FAS). The exposed group shows reduced facial growth relative to controls. (B) Comparison across the three groups. The AE‐FAS group demonstrates significantly lower PC1 values compared with controls (*p* < 0.001), whereas the AE group does not significantly differ from controls (*p* = 0.15). (C) Female‐only analysis showing differences in PC1 between AE and control groups. The AE‐FAS group is shown for descriptive purposes only due to the limited sample size. (D) Male‐only analysis showing differences in PC1 between AE and control groups, which are less pronounced than those observed in females. Lines represent linear regression fits across age. Statistical comparisons were performed between the AE and control groups only. The AE‐FAS group, particularly in females (*n* = 6), is displayed for descriptive purposes and was not included in statistical testing due to limited sample size.

Dense correspondence across more than 25,000 surface points per face was established using the 20 annotated anatomical landmarks, enabling the construction of DSMs of facial morphology. In DSMs constructed from mixed‐age samples, PC1 primarily captured variation related to overall facial growth. We first combined the AE and AE‐FAS groups into a single exposed category and compared this group with controls (Figure 3). The exposed group showed significantly reduced facial growth relative to controls (*p* < 0.0001). When the two diagnostic subgroups were examined separately, the AE‐FAS group exhibited markedly reduced facial growth compared with controls (*p* < 0.0001), whereas no significant difference was observed between the AE group and controls (*p* = 0.72). These analyses included age, sex, and BMI *z* scores as covariates.

**FIGURE 4:**
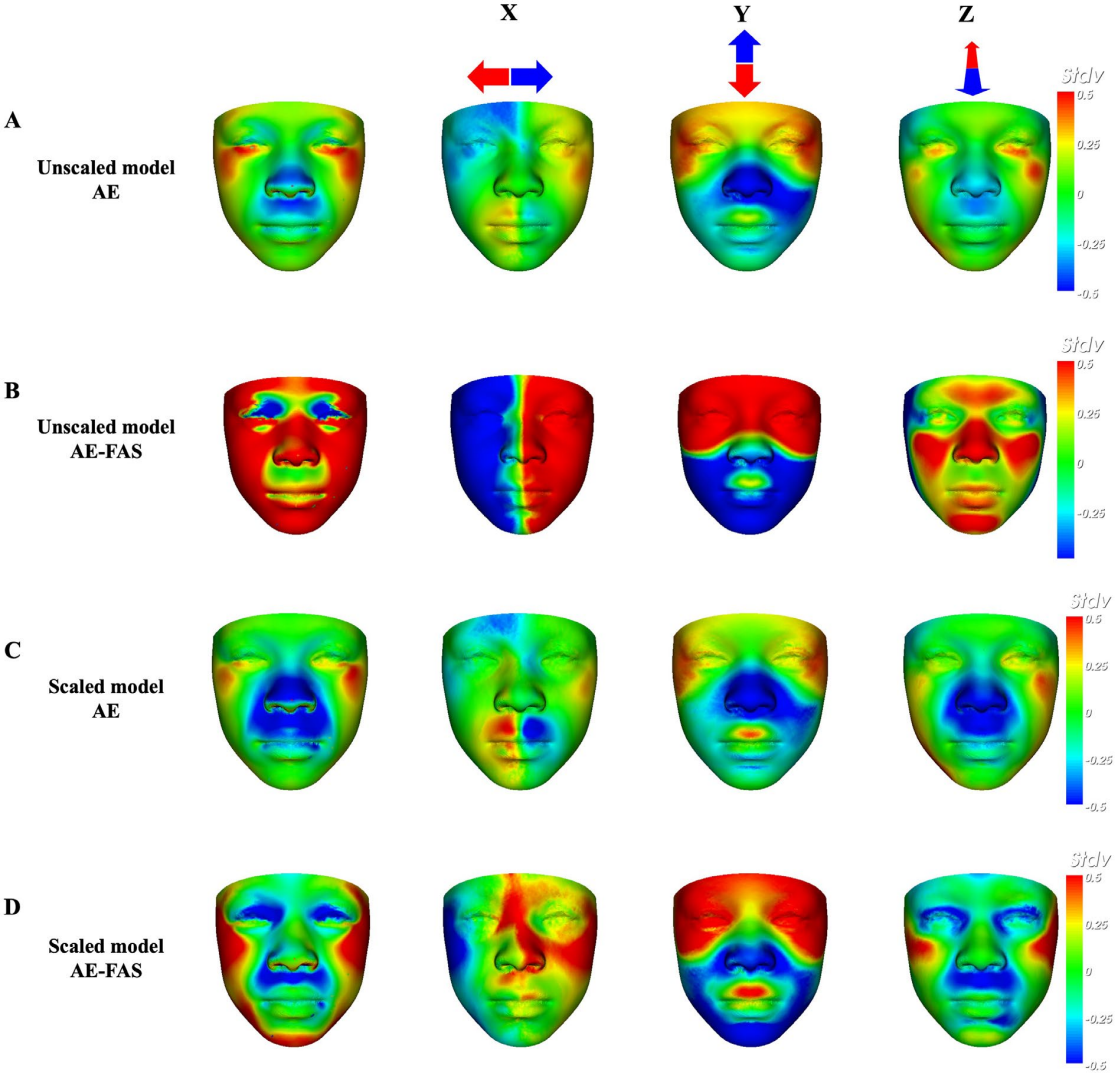
Comparison of mean facial signatures along each spatial axis between alcohol‐exposed groups and controls. (A) Mean facial signature of the AE group and (B) mean facial signature of the AE‐FAS group derived from the unscaled model; (C) Mean facial signature of the AE group and (D) mean facial signatures of the AE‐FAS group derived from the size‐scaled (shape‐only) model. All facial signatures were generated by normalizing group mean faces against 35 age‐matched control faces. Red–green–blue heatmaps represent contraction–coincidence–expansion along the surface normal relative to age‐matched controls. The displayed color scale corresponds to ±0.5 SD, with the extreme blue and red colors indicating the upper and lower bounds of the scale. In the lateral axis, opposing red–blue displacement patterns around the inner and outer canthi indicate shortened palpebral fissures. In the vertical axis, blue shading over the nasal region reflects reduced nasal length and superior displacement. In the depth (anterior–posterior) axis, red regions across the malar and zygomatic areas indicate midfacial flattening. Blue shading over the philtrum denotes philtral smoothing, whereas red shading over the chin corresponds to relative mandibular retrusion.

Growth deficiency is a core feature of FASD. To disentangle size‐related effects from shape‐specific dysmorphology, we constructed both combined size‐and‐shape DSMs and shape‐only DSMs. In the unscaled models, the pronounced reduction in overall facial size in the AE‐FAS group often dominated the visualizations, resulting in near‐uniform heat maps that obscured more subtle regional shape differences. To address this, all facial surfaces were scaled to a common reference template prior to constructing DSMs, thereby removing size variation and enabling shape‐only comparisons. Both size‐and‐shape and size‐normalized average faces for the AE‐FAS and AE groups are shown in Figure 4.

The resulting shape‐only facial signatures revealed characteristic patterns of dysmorphology across the three spatial axes. In the lateral axis, opposing red–blue displacement patterns around the inner and outer canthi indicate shortened palpebral fissures. Vertical displacement maps show reduced nasal length and upward displacement of the nasal region, while depth (anterior–posterior) maps highlight malar flattening, upper lip protrusion, and relative mandibular retrusion. The combination of upper lip protrusion and malar flattening suggests a rotational pattern involving the premaxillary region. These features were more pronounced in the AE‐FAS group, whereas corresponding patterns in the AE group were weaker and required lower significance thresholds for visualization.

Quantitative comparisons using FSW and MSW indicated significant differences between controls and AE‐FAS in the size‐and‐shape model (FSW: *p* = 0.0023; MSW: *p* = 0.0002), whereas no significant differences were observed after size normalization (all *p* > 0.05).

Finally, DSMs were also constructed for selected facial subregions, including the eyes, malar region, nose, lip–philtrum region, profile, and zygomatic arch. Mean facial signatures for these subregional analyses are presented in the Figure S3.

### 3.4 Sex‐Stratified Analyses of Facial Morphology

To evaluate potential sex‐specific effects of PAE, we conducted sex‐stratified analyses in males and females separately. Owing to the limited sample size in the AE‐FAS subgroup, comparisons were restricted to controls and the AE group within each sex, adjusting for age and BMI *z* scores.

Facial growth was first assessed using PC1 derived from the unscaled model. The association between PAE and reduced facial growth was substantially stronger in females than in males (Figure 3), as reflected by larger effect sizes (partial *η* ^2^ = 0.13 vs. 0.03).

We next examined individual facial measurements using both unscaled and scaled models. Among the 15 unscaled traits, five showed significant differences between controls and AE females (ICD, OCD, CI, PL, MAL), whereas only one measurement (MAL) was significant in males. Overall, group differences were more pronounced in females, consistent with stronger statistical evidence in this group.

To account for overall size effects, we further analyzed size‐normalized measurements representing shape‐only variation. In females, five traits remained significant after normalization, including PL and CI (significant in both unscaled and scaled models), as well as TFH, LFH, and MV. In contrast, only MAL remained significant in males after normalization. Notably, MAL was no longer significant in females after normalization. Detailed results of the sex‐stratified analyses are presented in Table 3.

**TABLE 3:** Sex‐stratified ANCOVA results for facial measurements.

Variables	Female			Male		
	Control	AE	*p*	Control	AE	*p*
Unscaled measurements						
LPFL	28.0 ± 2.3	27.6 ± 1.5	0.22	28.1 ± 2.0	27.6 ± 1.8	0.17
RPFL	28.4 ± 2.3	28.0 ± 1.8	0.18	28.5 ± 1.9	28.1 ± 1.6	0.18
PFL	28.2 ± 2.2	27.8 ± 1.6	0.18	28.3 ± 1.8	27.9 ± 1.6	0.14
ICD	35.4 ± 3.0	33.6 ± 2.3	0.0007***	35.7 ± 3.7	35.8 ± 3.3	0.83
OCD	89.3 ± 6.1	87.0 ± 4.0	0.01*	90.5 ± 5.9	89.6 ± 4.9	0.33
CI	0.40 ± 0.02	0.39 ± 0.02	0.02*	0.39 ± 0.03	0.40 ± 0.02	0.32
NH	49.5 ± 4.6	48.5 ± 4.0	0.19	49.2 ± 4.6	49.4 ± 5.3	0.74
NL	41.9 ± 4.4	41.0 ± 4.4	0.21	41.7 ± 4.7	41.4 ± 5.4	0.75
NP	12.1 ± 2.0	11.9 ± 1.9	0.46	12.8 ± 2.3	12.6 ± 2.5	0.69
PL	13.5 ± 2.8	14.7 ± 2.2	0.02*	15.7 ± 2.8	16.1 ± 2.8	0.41
NHR	0.46 ± 0.02	0.45 ± 0.02	0.37	0.44 ± 0.02	0.44 ± 0.02	0.32
TFH	108.9 ± 9.0	107.6 ± 7.8	0.28	112.7 ± 10.2	111.9 ± 11.2	0.60
LFH	61.6 ± 5.6	61.7 ± 5.4	0.83	66.0 ± 7.4	65.5 ± 7.7	0.77
MV	65321.0 ± 15904.3	62656.2 ± 13695.8	0.14	73406.5 ± 20239.5	70568.6 ± 19862.8	0.27
MAL	253.4 ± 23.3	244.0 ± 21.6	0.0005***	255.8 ± 24.0	250.0 ± 19.8	0.01*
Scaled measurements						
LPFL	26.4 ± 1.3	26.8 ± 1.5	0.19	26.0 ± 1.6	25.8 ± 1.7	0.48
RPFL	26.8 ± 1.3	27.1 ± 1.5	0.25	26.4 ± 1.5	26.2 ± 1.6	0.59
PFL	26.6 ± 1.2	26.9 ± 1.5	0.18	26.2 ± 1.4	26.0 ± 1.6	0.49
ICD	33.3 ± 2.2	32.6 ± 2.1	0.07	33.0 ± 2.6	33.3 ± 2.6	0.45
OCD	84.3 ± 2.8	84.5 ± 3.1	0.73	83.6 ± 3.4	83.6 ± 4.0	1.00
CI	0.40 ± 0.02	0.39 ± 0.02	0.02*	0.39 ± 0.03	0.40 ± 0.02	0.32
NH	46.6 ± 2.8	47.0 ± 3.1	0.51	45.4 ± 3.0	45.9 ± 3.6	0.36
NL	39.4 ± 2.9	39.7 ± 3.1	0.70	38.5 ± 3.1	38.5 ± 4.0	0.88
NP	11.3 ± 1.4	11.5 ± 1.4	0.65	11.7 ± 1.6	11.7 ± 1.8	0.93
PL	12.7 ± 2.3	14.2 ± 2.1	0.001**	14.5 ± 2.3	14.9 ± 2.2	0.30
NHR	0.46 ± 0.02	0.45 ± 0.02	0.37	0.44 ± 0.02	0.44 ± 0.02	0.32
TFH	102.6 ± 3.9	104.3 ± 4.3	0.04*	103.9 ± 4.1	104.0 ± 6.2	0.80
LFH	58.0 ± 3.0	59.8 ± 3.6	0.01*	60.8 ± 3.7	60.9 ± 4.6	0.74
MV	53719.9 ± 5628.9	56384.2 ± 7005.5	0.02*	56511.8 ± 6805.0	55494.6 ± 7370.6	0.48
MAL	238.6 ± 8.2	235.8 ± 9.8	0.08	235.8 ± 8.4	232.5 ± 8.5	0.02*

*Note:* Unscaled measurements represent original facial dimensions incorporating both size and shape variation, whereas scaled measurements were normalized for overall facial size to capture shape‐only variation. This comparison is for control and AE group only. Values are mean ± SD. *p < 0.05; **p < 0.01; ***p < 0.001.

Abbreviations: CI, canthal index; ICD, inner canthal distance; LFH, lower facial height; LPFL, left palpebral fissure length; MAL, mandibular arc length; MV, mandibular volume; NH, nasal height; NHR, nasal height ratio; NL, nasal length; NP, nasal protrusion; OCD, outer canthal distance; PFL, mean palpebral fissure length; PL, philtrum length; RPFL, right palpebral fissure length; TFH, total facial height.

Formal group‐by‐sex interaction analyses were subsequently performed. Although one measurement (CI) showed nominal evidence of a group‐by‐sex interaction (*p* = 0.024), no interaction remained significant after correction for multiple testing (Table S5).

### 3.5 Facial Signature Graphs of Facial Dysmorphism Across the Fetal Alcohol Spectrum

Signature graph cluster analysis was performed on 21 individuals with AE‐FAS and 77 individuals categorized as AE, providing an integrated visualization of phenotypic variation across the fetal alcohol spectrum. In the resulting graph, each node represents an individual facial signature, and edges connect each node to its nearest morphological neighbor, forming clusters of phenotypically similar faces.

**FIGURE 5:**
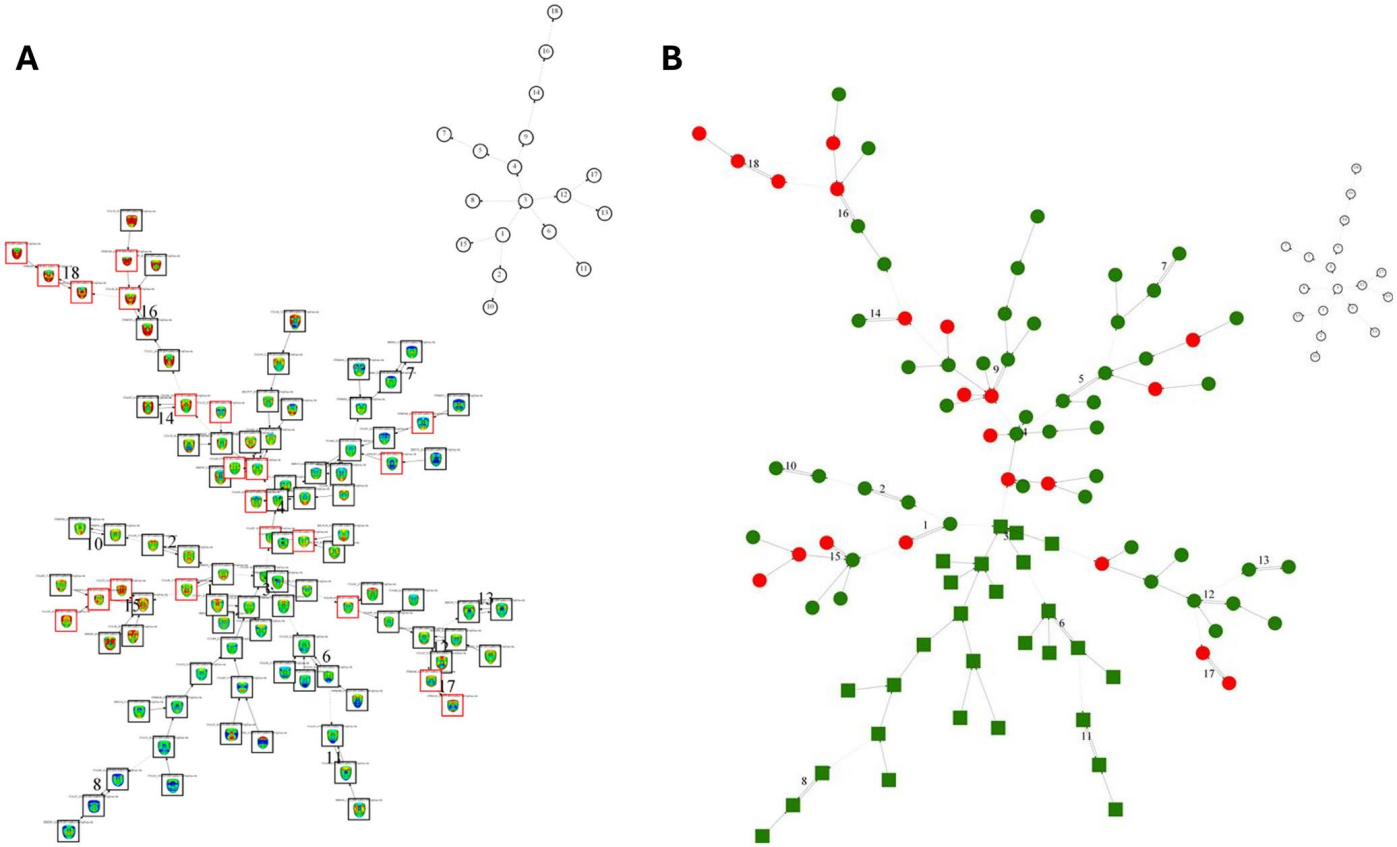
Face signature graphs of 98 alcohol‐exposed individuals. (A) Face signature graph of 18 clusters arising from normalization against controls using the unscaled model for 98 children classified as AE or AE‐FAS. Each node represents an individual, and edges connect individuals with similar facial signatures. Magnified insets highlight local regions of the graph to illustrate connections among individuals with highly similar facial patterns. (B) Color‐coded representation of the graph shown in panel A. Nodes are colored by diagnostic group: Red indicates AE‐FAS individuals, and green indicates AE individuals. Square nodes denote individuals belonging to a distinct subgroup composed exclusively of AE individuals. This subgroup represents a non‐FAS‐like cluster, whereas AE individuals located outside this subgroup and in closer proximity to AE‐FAS nodes exhibit more FAS‐like facial characteristics.

In the color‐coded representation (Figure 5), red nodes correspond to AE‐FAS individuals, whereas green nodes represent AE individuals without FAS‐associated physical features. A distinct subgroup composed exclusively of AE individuals is identifiable within the graph and is indicated by square nodes. This subgroup corresponds to a non‐FAS‐like cluster, defined as the largest connected component of AE individuals that does not include or connect to any AE‐FAS nodes.

In contrast, AE individuals located outside this subgroup, particularly those within or connected to components containing AE‐FAS nodes, exhibit facial signatures more similar to those of the AE‐FAS group and were therefore classified as FAS‐like.

Together, these patterns highlight substantial heterogeneity within the AE group and demonstrate that facial morphology varies along a continuum, with a subset of clinically non‐FAS individuals showing phenotypic affinity to the AE‐FAS group.

### 3.6 Data‐Driven Subgrouping of AE Faces

**FIGURE 6:**
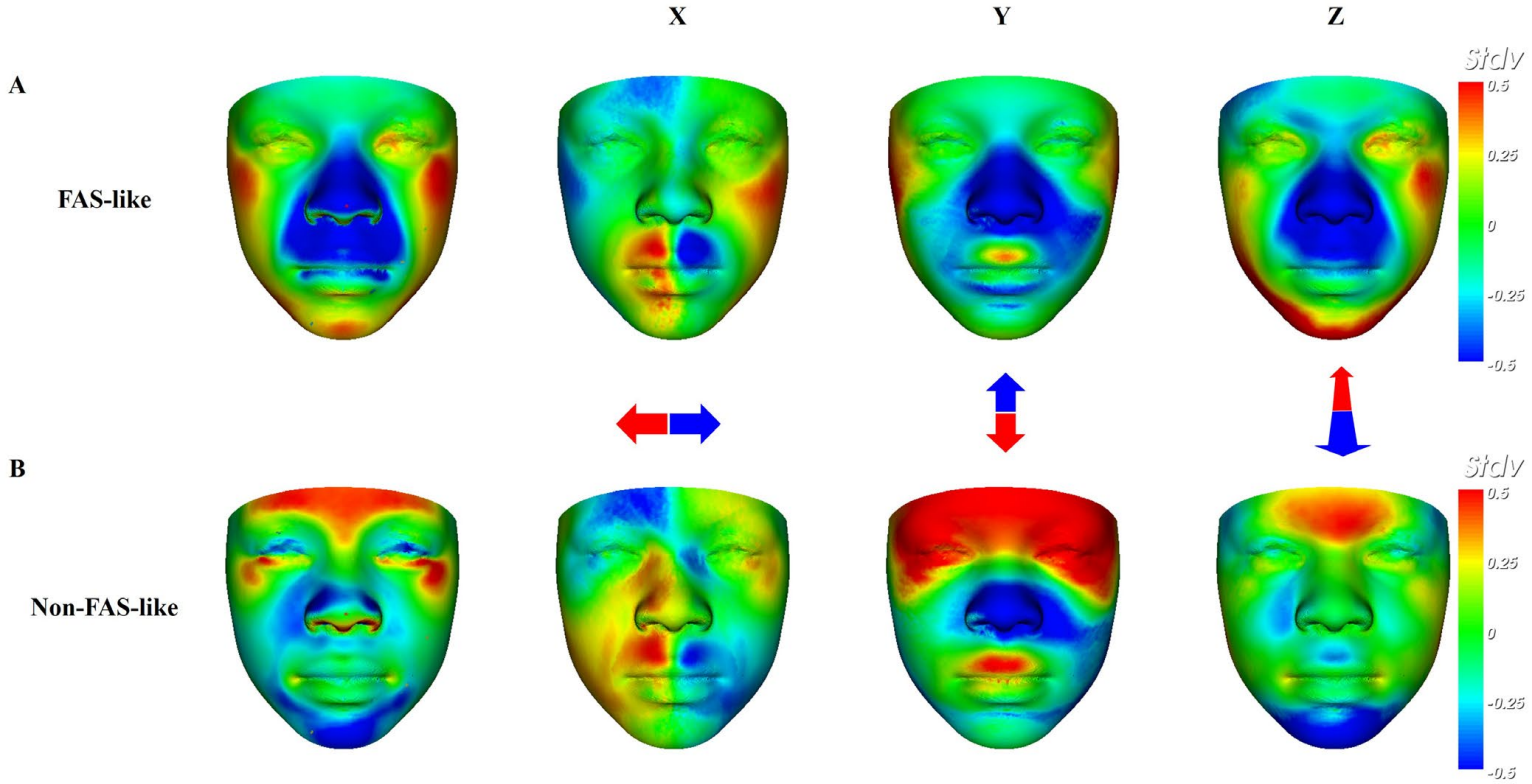
Mean facial signatures for FAS‐like and non‐FAS‐like subgroups across spatial axes. (A) FAS‐like and (B) non‐FAS‐like mean facial signatures derived from the size‐scaled (shape‐only) model. Signatures are shown along the lateral (left–right), vertical (superior–inferior), and depth (anterior–posterior) axes. Red–green–blue heatmaps indicate contraction–coincidence–expansion relative to age‐matched controls at the indicated significance thresholds.

Using facial signature‐graph clustering, the AE group was stratified into two phenotypic subgroups: FAS‐like (*n* = 49) and non‐FAS‐like (*n* = 28). Subgroup‐specific average facial signatures revealed distinct patterns of facial morphology. The FAS‐like subgroup exhibited a more pronounced dysmorphic pattern, recapitulating key facial characteristics observed in the AE‐FAS group and closely resembling the combined alcohol‐exposed average shown in Figure 4. In contrast, the non‐FAS‐like subgroup displayed comparatively milder deviations from the control reference (Figure 6).

To quantitatively assess these differences, we performed an ANCOVA across four groups (controls, AE‐FAS, FAS‐like, and non‐FAS‐like), adjusting for age, sex, and BMI *z* score. Among unscaled measurements, 13 of 15 traits showed significant overall group effects. Relative to controls, the FAS‐like subgroup exhibited widespread reductions in facial dimensions, closely paralleling the pattern observed in the AE‐FAS group, whereas the non‐FAS‐like subgroup showed more limited and generally intermediate differences (Table S6).

Direct comparison between the FAS‐like and non‐FAS‐like subgroups identified only two unscaled traits with significant differences: OCD (*p* = 0.0405) and MAL (*p* < 0.0001). For the scaled measurements, several traits showed overall group effects (LFH, PL, NP, and MAL); however, only MAL remained significantly different between the FAS‐like and non‐FAS‐like subgroups (Table S6).

### 3.7 Neurobehavioral Comparisons of Facial Subgroups

To determine whether the facial subtypes identified through data‐driven clustering were associated with differences in neurobehavioral functioning, neurobehavioral data were analyzed for 215 participants across four groups: AE‐FAS, FAS‐like, non‐FAS‐like, and controls. A total of 33 neurobehavioral variables were analyzed, representing key functional domains including executive function, behavioral and emotional problems, memory performance, spatial working memory, adaptive functioning, and attention‐related symptoms (Table S7). Sample sizes varied slightly across measures due to missing data; for each analysis, only individuals with complete data for the outcome and covariates were included. Group sizes for each variable are provided in Table S8. Group differences were assessed using ANCOVA, adjusting for age, sex, and BMI *z* score. To account for multiple comparisons across the large number of neurobehavioral measures, *p* values were corrected using the FDR method.

Consistent with expectations, children with AE‐FAS exhibited significant impairments across multiple neurobehavioral domains compared with controls, including executive function, behavioral problems, and working memory performance. In contrast, differences between the FAS‐like and non‐FAS‐like subgroups were limited. Across all measures examined, only one trait—DBD ADHD inattentive symptoms remained significantly different between the two subgroups after multiple testing correction (FDR‐adjusted *p* = 0.03). No other neurobehavioral measures showed significant differences between these two subgroups after correction (Table S8).

### 3.8 Discriminant Analysis

Discriminant analyses were performed using both the full‐face DSM and selected facial subregions. Twenty randomly sampled 90% training and 10% unseen test‐set pairs yielded a mean area under the receiver operating characteristic curve (AUC) of 0.778 for the full‐face model, corresponding to the probability that the model would correctly distinguish between a randomly selected control face and a randomly selected AE‐FAS face (Table 4). Analyses based on facial subregions yielded modest improvements in discrimination performance. Models focusing on the lip‐philtrum and periorbital regions achieved the highest accuracy (AUC = 0.870; Table 4).

**TABLE 4:** Agreement between facial classification and clinical categorization.

	CM	LDA	SVM
Eyes	0.846	0.846	0.835
Malar	0.843	0.843	0.836
Lip‐philtrum	0.879	0.857	0.875
Zygomatic arch	0.843	0.843	0.832
Nose	0.807	0.843	0.846
Thin profile	0.807	0.825	0.828
Full face	0.757	0.764	0.778

*Note:* Each classification rate was calculated as the average area under the ROC curve across 20 cross‐validation trials, representing the likelihood of correctly distinguishing between two individuals, one from each of the groups being compared. Analyses were performed using the full face and selected facial regions, including the eye, malar, lip–philtrum, zygomatic arch, nose, and profile.

Abbreviations: CM, closest mean; LDA, linear discriminant analysis; SVM, support vector machines.

## 4 Discussion

In this study, we applied Dense Surface Modeling and facial signature analysis to characterize facial morphology associated with PAE in African‐American children, a population that has been historically underrepresented in FASD research (Ergun et al. 2021; Rockhold et al. 2024). Overall, our findings are consistent with prior studies demonstrating that growth‐related effects represent a dominant component of alcohol‐associated facial dysmorphology (Suttie et al. 2013, 2017, 2018). Across analyses, reductions in global facial growth emerged as the most robust signal, aligning with previous work using DSM‐based approaches in South African and European cohorts, which similarly identified facial growth restriction as a core feature of the FASD facial phenotype (Suttie et al. 2013, 2018).

While global growth reduction represents the dominant signal, it does not fully explain the regional complexity of facial dysmorphology. Because some variation in facial morphology is driven by growth and facial size, we normalized these effects to analyze facial shapes. This approach allowed us to compare the shape of specific facial regions independent of global growth differences. Consequently, the observed morphological differences are more likely to reflect effects associated with PAE rather than generalized growth variation. Within this growth‐normalized framework, several key findings emerged. First, facial and growth‐related measures demonstrated a continuum across groups, with controls generally showing the largest mean values, children with PAE and FAS‐associated facial features the smallest, and children with PAE without FAS features falling intermediate between them. This pattern suggests that PAE may influence facial morphology and growth even in the absence of the characteristic facial features required for an FAS diagnosis. Second, sex‐stratified analyses demonstrated distinct patterns of facial dysmorphology between males and females, suggesting potential sex‐specific sensitivity to PAE. Females demonstrated significant reductions in overall facial growth and reductions in ocular measurements, together with increased philtrum length. After controlling for global facial size to evaluate shape‐specific differences, several facial differences remained observable among females, including reduced canthal index and increased philtrum length and upper facial height. In contrast, males demonstrated a more restricted pattern of shape‐related dysmorphology, with a reduced mandibular arc measurement representing micrognathia as the primary significant feature. Collectively, these findings indicate sex‐stratified differences in the pattern of PAE‐associated facial morphology in this cohort, with broader growth‐related and shape‐specific signals observed in females, and more localized mandibular alterations observed in males. Third, analyses within children with PAE revealed substantial heterogeneity in facial phenotypes, with a subset of AE individuals exhibiting facial morphology that more closely resembled those with FAS, supporting the presence of a continuum of alcohol‐related facial dysmorphology across the FASD spectrum. Notably, facial differences were observed even among individuals who did not meet clinical criteria for FAS based on cardinal facial features. This finding suggests that subtle, subclinical manifestations of alcohol‐related dysmorphology may exist beyond current diagnostic thresholds, and that existing diagnostic classification frameworks may not fully capture the full spectrum of facial variation associated with PAE. However, despite the identification of distinct facial subgroups, these subgroupings did not show clear correspondence with neurobehavioral outcomes. This finding contrasts with previous observations in a mixed‐ancestry South African cohort, in which facial morphology‐based subgrouping corresponded more closely with neurobehavioral performance (Suttie et al. 2013). The absence of such correspondence in the present African‐American cohort may reflect differences in cohort composition, ascertainment strategies, or other study‐specific factors influencing the relationship between facial morphology and neurobehavioral impairment. More broadly, these findings suggest that facial dysmorphology alone may not reliably predict neurobehavioral severity in this population. Comprehensive assessment integrating both facial phenotyping and neurobehavioral evaluation therefore remains essential for accurate characterization of PAE‐related outcomes.

An important consideration when interpreting these findings is that classification of the AE‐FAS group incorporated established FAS‐associated facial features, including shortened palpebral fissure length, smooth philtrum, and thin upper vermilion border. Consequently, some degree of circularity is unavoidable when interpreting differences in these specific diagnostic traits between AE‐FAS participants and controls. However, the primary aim of the present study was not to reevaluate the diagnostic features used for group assignment, but rather to characterize broader facial morphology, facial shape variation, and additional facial characteristics beyond those incorporated into clinical classification. Many of the facial differences identified in this study were not used in determining AE‐FAS status and therefore provide information extending beyond the diagnostic criteria themselves.

At the level of specific anatomical features, several localized signals emerged. A novel contribution of this work is the use of 3D mandibular arc measurements derived from dense facial correspondence. Notably, mandibular arc differences remained significant after controlling for overall facial size, indicating that this feature reflects shape‐specific dysmorphology (micrognathia) rather than generalized growth deficiency. Mandibular involvement in FASD has been less frequently quantified in prior facial studies, which have focused predominantly on midfacial and periorbital features (Gomez et al. 2020; Suttie et al. 2013; Tsang et al. 2023). Our findings suggest that mandibular morphology may provide additional discriminatory information, particularly when size effects are removed, and highlight the value of 3D approaches for capturing complex craniofacial geometry.

In addition to mandibular changes, we identified previously underreported signals in other facial regions. We observed a pattern involving the zygomatic arch, characterized by altered surface displacement in both depth and lateral dimensions. To our knowledge, surface‐based evidence of zygomatic arch involvement in human FASD cohorts has not been previously reported, although alterations in this region have been described in animal models, including mouse studies examining alcohol‐induced craniofacial malformations (Padmanabhan and Muawad 1985). Given that surface‐based analyses of facial morphology in African‐American populations have not been conducted previously, this finding may represent a previously underappreciated aspect of alcohol‐related facial dysmorphology in this population. One possible mechanistic explanation involves disruption of developmental signaling pathways involved in craniofacial patterning, such as the Sonic hedgehog (SHH) pathway. SHH signaling plays a central role in midline patterning, neural crest cell migration, and craniofacial skeletal development, and experimental studies have shown that PAE can interfere with SHH signaling during embryogenesis (Ahlgren et al. 2002; Cartwright and Smith 1995; Dunty Jr. et al. 2001). Perturbations in neural crest‐derived structures could therefore plausibly contribute to the zygomatic and premaxillary rotational effects observed in our shape‐only models. Further studies integrating genetic and developmental data will be required to clarify the mechanisms underlying these morphological changes.

Beyond these morphological findings, our results also have important clinical implications. An important consideration when assessing individuals from diverse ancestral backgrounds is the use of population‐appropriate facial norms. Shortened PFL is a cardinal facial feature of FAS; however, the normative charts commonly applied in the clinical assessment of African‐American children are largely derived from Caucasian populations. Given well‐established ethnic differences in baseline palpebral fissure dimensions, reliance on Caucasian‐based reference standards is likely to underestimate the degree of palpebral fissure shortening in African‐American children, thereby increasing the risk of under‐identification of FAS‐related facial features. Our findings, together with prior reports documenting ethnic variation in ocular morphology, underscore the need for ancestry‐specific normative data and caution against the use of non‐matched reference charts in diagnostic decision‐making.

To explore the extent to which these facial features distinguish AE‐FAS participants from controls, we further assessed classification performance. Discriminant performance between controls and the AE‐FAS group was moderate. This likely reflects both the relatively small size of the AE‐FAS subgroup and substantial within‐group variability in facial morphology, consistent with the view that FASD‐related facial features form a continuous and heterogeneous phenotype rather than a single uniform facial pattern. Improved classification performance observed in specific facial subregions further suggests that group‐discriminating facial information may be regionally concentrated and partially diluted in full‐face models by normal population‐level variation. The lower discrimination accuracy in this cohort is also consistent with previous work using three‐dimensional facial analysis, which reported reduced classification performance in African‐American populations compared with other ancestral groups, with accuracies of approximately 70%–80% (Moore et al. 2007). However, these estimates should be interpreted with caution, as Moore et al. modeled only two features (PFL and philtrum length) in a small sample (11 FAS cases and 13 controls). The restricted feature set and sample size likely limit the reliability and generalizability of these findings. More broadly, reduced classification performance in this population may partly reflect the higher genetic admixture and phenotypic diversity observed in African‐American populations (Bryc et al. 2015; Parra et al. 1998), which could increase baseline facial morphological variability and reduce the separability of alcohol‐related facial features. In addition, all participants in this cohort self‐identified as African‐American, and racial identity is a socially constructed and heterogeneous classification that does not map directly onto genetic ancestry or facial morphology. Variation in self‐identification, particularly among individuals with mixed ancestry or diverse phenotypic presentations, may introduce additional unmeasured sources of variability, further attenuating classification accuracy. While a full exploration of these factors is beyond the scope of the present study, they are important considerations for interpreting model performance across socially defined population groups.

Certain facial regions may pose technical challenges for DSM, particularly when they are small and anatomically variable. The upper lip represents a relatively small anatomical region and may be more susceptible to noise in surface‐based analyses. To mitigate this, the upper lip vermilion and philtrum were combined to model the region more robustly, yielding good discrimination between controls and AE‐FAS (AUC = 0.88), exceeding that of other facial regions tested. Prior work has shown that discrimination in this region varies by population. Suttie et al. (2018) reported lower performance for upper lip vermilion analysis in a Caucasian cohort compared with a South African Cape Colored population (AUC 0.73 vs. 0.84). Similarly, in a recent study using OxSeg, a two‐dimensional approach for upper lip segmentation, improved discrimination accuracy was achieved in a similarly defined African‐American subpopulation compared to a white Caucasian cohort (98.6% vs. 92.5%, respectively) (Moghaddasi et al. 2025). This reaffirms that appearance‐based methods are preferable to morphological methods, where the visibility of the lip‐vermilion is paramount for accurately identifying the region. These results indicate that upper lip‐based discrimination performance is sensitive to both population characteristics and the analytical approach. In particular, methods that better capture visible features of the vermilion may outperform purely surface‐based morphological analyses in this region.

Despite the strengths of this study, several limitations should be acknowledged. The number of children meeting the criteria for AE‐FAS was relatively small, resulting in class imbalance (148 controls vs. 21 AE‐FAS), which may affect model performance. Standard classifiers were employed to investigate the variables' discriminative power and to assess whether they contain sufficient class‐separating information. Future work should focus on optimizing classifier performance, which was out of the scope of this study. In addition, AE‐FAS labeling in this study was based on physical features in conjunction with confirmed PAE rather than comprehensive diagnostic classification incorporating neurobehavioral criteria. While this approach is consistent with prior facial morphology studies and reflects real‐world diagnostic constraints, it may introduce some heterogeneity within diagnostic groups. Finally, incomplete information on the timing and dose of alcohol exposure limited our ability to examine exposure–dose–response relationships.

This study provides the first comprehensive surface‐based characterization of FASD‐related facial morphology in African‐American children. Our findings underscore the importance of ancestry‐appropriate, data‐driven approaches to FASD phenotyping. Future studies with larger cohorts, integrated neurobehavioral data, and longitudinal designs will be essential to refine these signatures, explore underlying developmental mechanisms, and ultimately improve diagnostic equity across populations.

## Funding

All or part of this work was done in conjunction with the Collaborative Initiative on Fetal Alcohol Spectrum Disorders (CIFASD, https://doi.org/10.5967/ntw9‐h991), which is funded by grants from the National Institute on Alcohol Abuse and Alcoholism (NIAAA). Research described in this paper was supported by NIAAA grant numbers: U01AA014809 (Suttie), U01AA026108 (Coles & Kable), U01AA026102 (Wozniak), U01AA014834 (Mattson), U01AA01480 (Wetherill), and U24AA014815 (del Campo). Additional information about CIFASD can be found at www.cifasd.org. This work was supported by the Chinese Academy of Medical Sciences (CAMS) Innovation Fund for Medical Science (CIFMS), China (grant number: 2024‐I2M‐2‐001‐1). This work was funded by the National Institutes of Health (NIH). The author (Q.L.) was supported by the Postdoctoral Fellowship Program of the China Postdoctoral Science Foundation (GZC20240143) and gratefully acknowledges financial support from the China Scholarship Council. The funders had no role in the design and conduct of the study; collection, management, analysis, and interpretation of the data; preparation, review, or approval of the manuscript; and decision to submit the manuscript for publication.

## Conflicts of Interest

The authors declare no conflicts of interest.

## Supporting information


**Table S1:** Diagnostic criteria used by the CIFASD dysmorphology core.
**Table S2:** Post hoc comparisons of sample characteristics between each pair of study groups.
**Table S3:** Results of ANCOVA for unscaled facial measurements after additional adjustment for recruitment site.
**Table S4:** Results of ANCOVA for facial measurements after excluding participants with mixed‐race ancestry.
**Table S5:** Group‐by‐sex interaction analyses for unscaled and scaled facial measurements across study groups (control, AE).
**Table S6:** ANCOVA and pairwise comparisons of unscaled and scaled facial measurements across study groups (control, AE‐FAS, FAS‐like, and non‐FAS‐like).
**Table S7:** Selected neurobehavioral measures and corresponding assessment instruments used in ANCOVA analyses.
**Table S8:** Pairwise comparisons from ANCOVA of selected neurobehavioral measures across diagnostic subgroups (AE‐FAS, FAS‐like, non‐FAS‐like, and controls).
**Figure S1:** Boxplot distributions of age, height, weight, and body mass index (BMI) *z* score across study groups.
**Figure S2:** Boxplot distributions of size‐normalized (scaled) philtrum length, nasal protrusion, and mandibular arc length across study groups.
**Figure S3:** Regional facial signatures derived from the size‐normalized (scaled) dense surface models for alcohol‐exposed subgroups relative to controls.
Methods S1:

## Data Availability

The data that support the findings of this study are available on request from the corresponding author. The data are not publicly available due to privacy or ethical restrictions.
