# Supplementary material for: Quantitative Three‐Dimensional Characterization of Facial Dysmorphism in Self‐Identified African‐American Individuals With Prenatal Alcohol Exposure
Source: Alcohol Clin Exp Res (Hoboken). 2026 Aug 7;50(8):e70396. doi: 10.1111/acer.70396 (PMC13451575; doi:10.1111/acer.70396)
Supplement: Supplementary file 1 [file ACER-50-0-s001.docx]

**Quantitative Three-Dimensional Characterization of Facial Dysmorphism in Self-Identified African American Individuals with Prenatal Alcohol Exposure**

**Table of Contents**

[Supplementary Methods	2](#_Toc232777059)

[Supplementary Table 1. Diagnostic criteria used by the CIFASD dysmorphology core	4](#_Toc232777060)

[Supplementary Table 2. Post-hoc comparisons of sample characteristics between each pair of study groups	6](#_Toc232777061)

[Supplementary Table 3. Results of ANCOVA for unscaled facial measurements after additional adjustment for recruitment site.	10](#_Toc232777062)

[Supplementary Table 4. Results of ANCOVA for facial measurements after excluding participants with mixed-race ancestry.	11](#_Toc232777063)

[Supplementary Table 5. Group-by-sex interaction analyses for unscaled and scaled facial measurements across study groups (control, AE).	12](#_Toc232777064)

[Supplementary Table 6. ANCOVA and pairwise comparisons of unscaled and scaled facial measurements across study groups (control, AE-FAS, FAS-like, and non–FAS-like)	14](#_Toc232777065)

[Supplementary Table 7. Selected neurobehavioral measures and corresponding assessment instruments used in ANCOVA analyses	17](#_Toc232777066)

[Supplementary Table 8. Pairwise comparisons from ANCOVA of selected neurobehavioral measures across diagnostic subgroups (AE-FAS, FAS-like, non–FAS-like, and controls)	20](#_Toc232777067)

[Supplementary Figure S1. Boxplot distributions of age, height, weight, body mass index (BMI) z score across study groups	24](#_Toc232777068)

[Supplementary Figure S2. Boxplot distributions of size-normalized (scaled) philtrum length, nasal protrusion and mandibular arc length across study groups	25](#_Toc232777069)

[Supplementary Figure S3. Regional facial signatures derived from the size-normalized (scaled) dense surface models for alcohol-exposed subgroups relative to controls	26](#_Toc232777070)

# Supplementary Methods

The following three-dimensional Euclidean distances were calculated based on manually annotated facial landmarks:

- **Left and right palpebral fissure length (LPFL and RPFL):** distance between the endocanthion and exocanthion on each side.
- **Mean palpebral fissure length (PFL):** average of left and right palpebral fissure lengths.
- **Inner canthal distance (ICD):** distance between the left and right endocanthion.
- **Outer canthal distance (OCD):** distance between the left and right exocanthion.
- **Canthal index (CI):** ratio of inner to outer canthal distance (ICD/OCD).
- **Nasal height (NH):** distance between nasion (N) and subnasale (Sn).
- **Nasal length (NL):** distance between nasion (N) and pronasale (Prn).
- **Nasal protrusion (NP):** shortest Euclidean distance from pronasale (Prn) to the line segment defined by nasion (N) and subnasale (Sn) in three-dimensional space.
  Let **NS = Sn − N** and **NP = Prn − N**.
  The projection parameter was computed as:

$$t=\frac{NP\cdot NS}{NS\cdot NS}$$

The parameter $t$was constrained to the interval [0,1] to ensure the closest point lay within the N–Sn segment.
The projected point was calculated as:

$$Q=N+t\cdot NS$$

Nasal protrusion was defined as the Euclidean distance between Prn and Q.

- **Philtrum length (PL):** distance between subnasale (Sn) and labiale superius (Ls).
- **Total facial height (TFH):** distance between nasion (N) and gnathion (Gn).
- **Lower facial height (LFH):** distance between subnasale (Sn) and gnathion (Gn).
- **Nasal height ratio (NHR):** ratio of nasal height to total facial height (N–Sn / N–Gn).
- **Mandibular volume (MV):** volume of the tetrahedron defined by bilateral otobasion inferius, gnathion (Gn), and labium inferius.
- **Mandibular arc length (MAL):** curved length of the inferior mandibular border measured on the three-dimensional facial mesh.
  A cutting plane was constructed using bilateral otobasion inferius and gnathion. The intersection of this plane with the mandibular surface generated a cross-sectional contour. The resulting curve was projected onto a local coordinate system and filtered to retain the inferior mandibular border between the left ear, gnathion, and right ear. The mandibular arc length was calculated by summing the Euclidean distances between consecutive points along the reconstructed curve.

# Supplementary Table 1. Diagnostic criteria used by the CIFASD dysmorphology core

| Criterion | Definition |
| --- | --- |
| Growth deficiency | Weight and/or height ≤10^th^ percentile |
| Microcephaly | Head circumference ≤10^th^ percentile |
| Structural abnormality | At least two of the following key facial features: |
|  | Palpebral fissure length ≤10th percentile |
|  | Smooth philtrum (score of 4 or 5 on Lipometer) |
|  | Thin vermillion border (score of 4 or 5 on Lipometer) |
|  | |
| Category | Required criteria |
| FAS | Structural abnormality and growth deficiency OR |
|  | Structural abnormality and microcephaly OR |
|  | Structural abnormality and microcephaly and growth deficiency |
| Deferred | At least one key facial feature (as previously mentioned) OR |
|  | Microcephaly and growth deficiency OR |
|  | Microcephaly or growth deficiency AND at least one of the following additional features: |
|  | Railroad track configuration ears |
|  | Ptosis |
|  | Heart murmur |
|  | Decreased pronation/supination at elbows |
|  | Camptodactyly |
|  | Other joint contractures |
|  | Hockey stick upper palmar crease |
| Not FAS | Does not meet criteria for either FAS or deferred category |

CIFASD = The Collaborative Initiative on Fetal Alcohol Spectrum Disorders; FAS = fetal alcohol syndrome.

# Supplementary Table 2. Post-hoc comparisons of sample characteristics between each pair of study groups

Estimates represent adjusted mean differences between groups derived from analysis of covariance (ANCOVA) models, controlling for age, sex, and BMI z scores.

Unscaled measurements represent original facial dimensions incorporating both size and shape variation, whereas scaled measurements were normalized for overall facial size to capture shape-only variation.

P values were adjusted for multiple comparisons using Bonferroni correction.

Abbreviations: LPFL: left palpebral fissure length; RPFL: right palpebral fissure length; PFL: mean palpebral fissure length; ICD: inner canthal distance; OCD: outer canthal distance; CI: canthal index; NL: nasal length; NH: nasal height; NP: nasal protrusion; PL: philtrum length; NHR: nasal height ratio; TFH: total facial height; LFH: lower facial height; MV: mandibular volume; MAL: mandibular arc length.

| **Variable** | **group1** | **group2** | **Estimate** | **P after adjustment** |
| --- | --- | --- | --- | --- |
| Unscaled measurements |  |  |  |  |
| LPFL | AE | CONTROL | -0.20 | 1.00 |
|  | AE | AE-FAS | 0.82 | 0.14 |
|  | CONTROL | AE-FAS | 1.02 | 0.03 |
| RPFL | AE | CONTROL | -0.18 | 1.00 |
|  | AE | AE-FAS | 1.08 | 0.02 |
|  | CONTROL | AE-FAS | 1.26 | <0.01 |
| PFL | AE | CONTROL | -0.19 | 1.00 |
|  | AE | AE-FAS | 0.95 | 0.04 |
|  | CONTROL | AE-FAS | 1.14 | 0.01 |
| ICD | AE | CONTROL | -0.47 | 0.70 |
|  | AE | AE-FAS | 0.68 | 0.99 |
|  | CONTROL | AE-FAS | 1.14 | 0.26 |
| OCD | AE | CONTROL | -0.70 | 0.62 |
|  | AE | AE-FAS | 2.81 | 0.01 |
|  | CONTROL | AE-FAS | 3.51 | <0.001 |
| CI | AE | CONTROL | 0.00 | 1.00 |
|  | AE | AE-FAS | -0.01 | 1.00 |
|  | CONTROL | AE-FAS | 0.00 | 1.00 |
| NH | AE | CONTROL | 0.17 | 1.00 |
|  | AE | AE-FAS | 2.14 | 0.06 |
|  | CONTROL | AE-FAS | 1.98 | 0.07 |
| NL | AE | CONTROL | -0.02 | 1.00 |
|  | AE | AE-FAS | 2.16 | 0.04 |
|  | CONTROL | AE-FAS | 2.18 | 0.03 |
| NP | AE | CONTROL | 0.14 | 1.00 |
|  | AE | AE-FAS | 1.51 | <0.001 |
|  | CONTROL | AE-FAS | 1.37 | <0.001 |
| PL | AE | CONTROL | 0.98 | 0.02 |
|  | AE | AE-FAS | 0.68 | 0.89 |
|  | CONTROL | AE-FAS | -0.30 | 1.00 |
| NHR | AE | CONTROL | 0.00 | 1.00 |
|  | AE | AE-FAS | 0.00 | 1.00 |
|  | CONTROL | AE-FAS | 0.00 | 1.00 |
| TFH | AE | CONTROL | 0.52 | 1.00 |
|  | AE | AE-FAS | 5.66 | <0.001 |
|  | CONTROL | AE-FAS | 5.14 | <0.01 |
| LFH | AE | CONTROL | 0.88 | 0.45 |
|  | AE | AE-FAS | 3.73 | <0.01 |
|  | CONTROL | AE-FAS | 2.86 | 0.02 |
| MV | AE | CONTROL | 157.26 | 1.00 |
|  | AE | AE-FAS | 6444.43 | 0.06 |
|  | CONTROL | AE-FAS | 6287.17 | 0.05 |
| MAL | AE | CONTROL | -3.60 | 0.10 |
|  | AE | AE-FAS | 4.73 | 0.36 |
|  | CONTROL | AE-FAS | 8.33 | 0.01 |
| Scaled measurements |  |  |  |  |
| LPFL | AE | CONTROL | -0.03 | 1.00 |
|  | AE | AE-FAS | 0.07 | 1.00 |
|  | CONTROL | AE-FAS | 0.10 | 1.00 |
| RPFL | AE | CONTROL | -0.01 | 1.00 |
|  | AE | AE-FAS | 0.33 | 1.00 |
|  | CONTROL | AE-FAS | 0.34 | 0.98 |
| PFL | AE | CONTROL | -0.02 | 1.00 |
|  | AE | AE-FAS | 0.20 | 1.00 |
|  | CONTROL | AE-FAS | 0.22 | 1.00 |
| ICD | AE | CONTROL | -0.26 | 1.00 |
|  | AE | AE-FAS | -0.28 | 1.00 |
|  | CONTROL | AE-FAS | -0.02 | 1.00 |
| OCD | AE | CONTROL | -0.18 | 1.00 |
|  | AE | AE-FAS | 0.39 | 1.00 |
|  | CONTROL | AE-FAS | 0.57 | 1.00 |
| CI | AE | CONTROL | 0.00 | 1.00 |
|  | AE | AE-FAS | -0.01 | 1.00 |
|  | CONTROL | AE-FAS | 0.00 | 1.00 |
| NH | AE | CONTROL | 0.40 | 0.99 |
|  | AE | AE-FAS | 0.76 | 0.91 |
|  | CONTROL | AE-FAS | 0.36 | 1.00 |
| NL | AE | CONTROL | 0.19 | 1.00 |
|  | AE | AE-FAS | 0.98 | 0.56 |
|  | CONTROL | AE-FAS | 0.79 | 0.79 |
| NP | AE | CONTROL | 0.20 | 0.84 |
|  | AE | AE-FAS | 1.14 | <0.01 |
|  | CONTROL | AE-FAS | 0.94 | 0.01 |
| PL | AE | CONTROL | 0.99 | 0.01 |
|  | AE | AE-FAS | 0.26 | 1.00 |
|  | CONTROL | AE-FAS | -0.73 | 0.59 |
| NHR | AE | CONTROL | 0.00 | 1.00 |
|  | AE | AE-FAS | 0.00 | 1.00 |
|  | CONTROL | AE-FAS | 0.00 | 1.00 |
| TFH | AE | CONTROL | 1.05 | 0.27 |
|  | AE | AE-FAS | 2.50 | 0.07 |
|  | CONTROL | AE-FAS | 1.45 | 0.51 |
| LFH | AE | CONTROL | 1.14 | 0.06 |
|  | AE | AE-FAS | 1.88 | 0.09 |
|  | CONTROL | AE-FAS | 0.73 | 1.00 |
| MV | AE | CONTROL | 1147.69 | 0.62 |
|  | AE | AE-FAS | 771.34 | 1.00 |
|  | CONTROL | AE-FAS | -376.35 | 1.00 |
| MAL | AE | CONTROL | -2.31 | 0.08 |
|  | AE | AE-FAS | -2.06 | 0.80 |
|  | CONTROL | AE-FAS | 0.25 | 1.00 |

# Supplementary Table 3. Results of ANCOVA for unscaled facial measurements after additional adjustment for recruitment site.

Results from sensitivity analyses in which recruitment site was included as an additional covariate in the ANCOVA models. Models were adjusted for age, sex, BMI z score, and recruitment site. Df1 and Df2 denote numerator and denominator degrees of freedom, respectively. F values and associated p values represent the overall effect of diagnostic group (Control, AE, and AE-FAS). The overall pattern of significant findings remained consistent with the primary analyses.

Abbreviations: LPFL: left palpebral fissure length; RPFL: right palpebral fissure length; PFL: mean palpebral fissure length; ICD: inner canthal distance; OCD: outer canthal distance; CI: canthal index; NL: nasal length; NH: nasal height; NP: nasal protrusion; PL: philtrum length; NHR: nasal height ratio; TFH: total facial height; LFH: lower facial height; MV: mandibular volume; MAL: mandibular arc length.

| Variable | Df1 | Df2 | F value | P value |
| --- | --- | --- | --- | --- |
| LPFL | 2 | 235 | 11.26 | 2.14E-05 |
| RPFL | 2 | 235 | 17.02 | 1.25E-07 |
| PFL | 2 | 235 | 15.94 | 3.23E-07 |
| ICD | 2 | 235 | 5.74 | 0.0037 |
| OCD | 2 | 235 | 21.80 | 2.06E-09 |
| CI | 2 | 235 | 0.69 | 0.50 |
| NH | 2 | 235 | 7.23 | 8.99E-04 |
| NL | 2 | 235 | 8.49 | 2.76E-04 |
| NP | 2 | 235 | 20.16 | 8.32E-09 |
| PL | 2 | 235 | 2.48 | 0.086 |
| NHR | 2 | 235 | 0.83 | 0.44 |
| TFH | 2 | 235 | 19.30 | 1.73E-08 |
| LFH | 2 | 235 | 13.04 | 4.27E-06 |
| MV | 2 | 235 | 15.74 | 3.84E-07 |
| MAL | 2 | 235 | 33.32 | 1.82E-13 |

# Supplementary Table 4. Results of ANCOVA for facial measurements after excluding participants with mixed-race ancestry.

Results from sensitivity analyses in which participants who reported mixed-race ancestry were excluded from the analytical sample. ANCOVA models were adjusted for age, sex, and BMI z score. Df1 and Df2 denote numerator and denominator degrees of freedom, respectively. F values and associated P values represent the overall effect of diagnostic group (Control, AE, and AE-FAS).

Abbreviations: LPFL: left palpebral fissure length; RPFL: right palpebral fissure length; PFL: mean palpebral fissure length; ICD: inner canthal distance; OCD: outer canthal distance; CI: canthal index; NL: nasal length; NH: nasal height; NP: nasal protrusion; PL: philtrum length; NHR: nasal height ratio; TFH: total facial height; LFH: lower facial height; MV: mandibular volume; MAL: mandibular arc length.

| Variable | Df1 | Df2 | F value | P value |
| --- | --- | --- | --- | --- |
| LPFL | 2 | 222 | 14.07 | 1.76E-06 |
| RPFL | 2 | 222 | 19.26 | 1.93E-08 |
| PFL | 2 | 222 | 18.84 | 2.77E-08 |
| ICD | 2 | 222 | 4.33 | 0.014 |
| OCD | 2 | 222 | 22.06 | 1.83E-09 |
| CI | 2 | 222 | 1.29 | 0.28 |
| NH | 2 | 222 | 6.45 | 0.0019 |
| NL | 2 | 222 | 7.97 | 4.54E-04 |
| NP | 2 | 222 | 23.11 | 7.63E-10 |
| PL | 2 | 222 | 1.34 | 0.26 |
| NHR | 2 | 222 | 0.93 | 0.4 |
| TFH | 2 | 222 | 19.42 | 1.68E-08 |
| LFH | 2 | 222 | 12.62 | 6.45E-06 |
| MV | 2 | 222 | 17.16 | 1.18E-07 |
| MAL | 2 | 222 | 35.49 | 4.22E-14 |

# Supplementary Table 5. Group-by-sex interaction analyses for unscaled and scaled facial measurements across study groups (control, AE).

ANCOVA models were fitted for each facial measurement with diagnostic group, sex, and a diagnostic group × sex interaction term, adjusting for age and BMI z-score. Male Effect and Female Effect represent the estimated diagnostic-group effects within males and females, respectively. F Interaction and P Interaction correspond to the formal test of the diagnostic group × sex interaction. False discovery rate (FDR) correction was applied separately across unscaled and scaled measurements.

Abbreviations: LPFL: left palpebral fissure length; RPFL: right palpebral fissure length; PFL: palpebral fissure length; ICD: inner canthal distance; OCD: outer canthal distance; CI: canthal index; NL: nasal length; NH: nasal height; NP: nasal protrusion; PL: philtrum length; NHR: nasal height ratio; TFH: total facial height; LFH: lower facial height; MV: mandibular volume; MAL: mandibular arc length.

| Variable | Male Effect | Female Effect | F Interaction | P Interaction | FDR-adjusted P |
| --- | --- | --- | --- | --- | --- |
| Unscaled measurements | | | | | |
| CI | -0.005 | 0.010 | 5.180 | 0.024 | 0.381 |
| ICD | -0.237 | 1.224 | 3.396 | 0.067 | 0.385 |
| NHR | -0.004 | 0.006 | 3.265 | 0.072 | 0.385 |
| PL | -0.447 | -1.583 | 2.531 | 0.113 | 0.452 |
| LFH | -0.097 | -1.822 | 2.031 | 0.156 | 0.470 |
| MV | 1681.070 | -2460.394 | 1.840 | 0.176 | 0.470 |
| PFL | 0.345 | 0.012 | 0.632 | 0.428 | 0.611 |
| LPFL | 0.367 | 0.013 | 0.589 | 0.443 | 0.611 |
| TFH | 0.019 | -1.239 | 0.586 | 0.445 | 0.611 |
| MAL | 4.677 | 2.176 | 0.560 | 0.455 | 0.611 |
| RPFL | 0.323 | 0.011 | 0.505 | 0.478 | 0.611 |
| NH | -0.510 | 0.185 | 0.474 | 0.492 | 0.611 |
| NP | -0.011 | -0.294 | 0.465 | 0.496 | 0.611 |
| OCD | 0.476 | 0.903 | 0.153 | 0.696 | 0.743 |
| NL | -0.092 | 0.114 | 0.045 | 0.833 | 0.833 |
| Scaled measurements | | | | | |
| MV | 805.943 | -3411.415 | 5.572 | 0.019 | 0.191 |
| CI | -0.005 | 0.010 | 5.180 | 0.024 | 0.191 |
| ICD | -0.371 | 0.952 | 3.900 | 0.050 | 0.202 |
| PL | -0.442 | -1.614 | 3.326 | 0.070 | 0.202 |
| NHR | -0.004 | 0.006 | 3.265 | 0.072 | 0.202 |
| LFH | -0.335 | -2.080 | 3.186 | 0.076 | 0.202 |
| TFH | -0.371 | -1.854 | 1.499 | 0.222 | 0.476 |
| MAL | 3.390 | 1.002 | 1.306 | 0.254 | 0.476 |
| PFL | 0.211 | -0.185 | 1.124 | 0.290 | 0.476 |
| LPFL | 0.235 | -0.189 | 1.091 | 0.297 | 0.476 |
| RPFL | 0.187 | -0.181 | 0.832 | 0.363 | 0.484 |
| NP | -0.053 | -0.359 | 0.720 | 0.397 | 0.489 |
| NH | -0.620 | -0.172 | 0.291 | 0.590 | 0.674 |
| OCD | 0.080 | 0.286 | 0.057 | 0.811 | 0.865 |
| NL | -0.224 | -0.152 | 0.008 | 0.931 | 0.931 |

# Supplementary Table 6. ANCOVA and pairwise comparisons of unscaled and scaled facial measurements across study groups (control, AE-FAS, FAS-like, and non–FAS-like)

ANCOVA models were performed for each facial measurement with diagnostic group included as the main effect and age, sex, and BMI z score included as covariates. Reported ANCOVA p values represent overall group effects. Pairwise p values represent post hoc comparisons between study groups with multiple-testing adjustment.

Unscaled measurements represent original facial dimensions incorporating both size and shape variation, whereas scaled measurements were normalized for overall facial size to capture shape-only variation.

Significance levels are indicated as * p < 0.05, ** p < 0.01, *** p < 0.001.

Abbreviations: LPFL: left palpebral fissure length; RPFL: right palpebral fissure length; PFL: palpebral fissure length; ICD: inner canthal distance; OCD: outer canthal distance; CI: canthal index; NL: nasal length; NH: nasal height; NP: nasal protrusion; PL: philtrum length; NHR: nasal height ratio; TFH: total facial height; LFH: lower facial height; MV: mandibular volume; MAL: mandibular arc length.

|  | ANCOVA  p value | control  v.s.  FAS | control  v.s.  FAS-like | control  v.s.  non-FAS-like | AE-FAS  v.s.  FAS-like | AE-FAS  v.s.  non-FAS-like | FAS-like v.s.  non-FAS-like |
| --- | --- | --- | --- | --- | --- | --- | --- |
| unscaled measurements | | | | | | | |
| LPFL | 4.63×10⁻⁵*** | 0.06 | 1 | 1 | 0.48 | 0.35 | 1 |
| RPFL | 8.11×10⁻⁸*** | 0.005** | 1 | 1 | 0.16 | 0.02* | 1 |
| PFL | 3.69×10⁻⁷*** | 0.009** | 1 | 1 | 0.21 | 0.06 | 1 |
| ICD | 4.05×10⁻⁴*** | 0.43 | 0.2 | 1 | 1 | 0.3 | 0.21 |
| OCD | 6.52×10⁻11*** | 0.0008*** | 0.13 | 1 | 0.23 | 0.002** | 0.13 |
| CI | 0.45 | 1 | 1 | 1 | 1 | 1 | 1 |
| NH | 0.00216** | 0.13 | 1 | 1 | 0.36 | 0.05 | 1 |
| NL | 0.00083*** | 0.05 | 1 | 1 | 0.28 | 0.04* | 1 |
| NP | 1.12×10⁻⁸*** | 0.0006*** | 1 | 1 | 0.0026** | 0.0003*** | 1 |
| PL | 0.047* | 1 | 0.59 | 0.05 | 1 | 0.82 | 1 |
| NHR | 0.57 | 1 | 1 | 1 | 1 | 1 | 1 |
| TFH | 1.64×10⁻⁸*** | 0.002** | 1 | 0.99 | 0.01* | 0.0008*** | 1 |
| LFH | 9.11×10⁻6*** | 0.04* | 1 | 1 | 0.009** | 0.02* | 1 |
| MV | 1.17×10⁻⁸*** | 0.08 | 1 | 0.82 | 0.56 | 0.02* | 0.32 |
| MAL | <2×10⁻¹⁶*** | 0.0091** | <0.0001*** | 0.19 | 1 | 0.0003*** | <0.0001*** |
| scaled measurements | | | | | | | |
| LPFL | 0.23 | 1 | 1 | 1 | 1 | 1 | 0.52 |
| RPFL | 0.62 | 1 | 1 | 1 | 1 | 1 | 1 |
| PFL | 0.38 | 1 | 1 | 1 | 1 | 1 | 0.91 |
| ICD | 0.78 | 1 | 1 | 1 | 1 | 1 | 1 |
| OCD | 0.8 | 1 | 1 | 1 | 1 | 1 | 1 |
| CI | 0.45 | 1 | 1 | 1 | 1 | 1 | 1 |
| NH | 0.31 | 1 | 1 | 1 | 1 | 1 | 1 |
| NL | 0.42 | 1 | 1 | 1 | 1 | 1 | 1 |
| NP | 0.00021*** | 0.01* | 1 | 1 | 0.007** | 0.01 | 1 |
| PL | 0.02* | 1 | 0.13 | 0.11 | 1 | 1 | 1 |
| NHR | 0.57 | 1 | 1 | 1 | 1 | 1 | 1 |
| TFH | 0.067 | 1 | 0.29 | 1 | 0.08 | 1 | 1 |
| LFH | 0.031 | 1 | 0.02* | 1 | 0.06 | 1 | 0.49 |
| MV | 0.76 | 1 | 1 | 1 | 1 | 1 | 1 |
| MAL | 3.55×10⁻⁸*** | 1 | 0.0003*** | 0.88 | 0.12 | 1 | 0.0003*** |

# Supplementary Table 7. Selected neurobehavioral measures and corresponding assessment instruments used in ANCOVA analyses

Neurobehavioral measures were selected to represent key functional domains, including executive function, behavioral and emotional problems, memory performance, spatial working memory, adaptive functioning, and ADHD-related symptoms.

Each domain was assessed using established instruments: the Behavior Rating Inventory of Executive Function (BRIEF), the Child Behavior Checklist (CBCL), the Cambridge Neuropsychological Test Automated Battery (CANTAB), including the Delayed Matching to Sample (DMS) and Spatial Working Memory (SWM) tasks, the Vineland Adaptive Behavior Scales, Second Edition (VABS-II), and the Disruptive Behavior Disorders (DBD) rating scale. These measures include both caregiver-reported questionnaires and performance-based cognitive tasks.

| **Functional Domain** | **Assessment Instrument** | **Measure** | **Description** |
| --- | --- | --- | --- |
| **Executive Function** | BRIEF | briefpgect | Global Executive Composite T-score (overall executive function) |
|  |  | briefpbrit | Behavioral Regulation Index T-score (inhibition, cognitive flexibility, and emotional control) |
|  |  | briefpmit | Metacognition Index T-score (working memory, planning, organization, and monitoring) |
| **Behavioral & Emotional Problems** | CBCL | cbcltotprobt | Total Problems T-score (overall behavioral and emotional problems) |
|  |  | cbclinternalprobt | Internalizing Problems T-score (anxiety, depression, withdrawal) |
|  |  | cbclexternalprobt | Externalizing Problems T-score (aggression, rule-breaking behavior) |
|  |  | cbcladhdt | ADHD Problems T-score |
|  |  | cbclanxdeprt | Anxious/Depressed T-score |
|  |  | cbclsomcomplt | Somatic Complaints T-score |
|  |  | cbclattprobt | Attention Problems T-score |
|  |  | cbclaggbehavt | Aggressive Behavior T-score |
|  |  | cbclsocprobt | Social Problems T-score |
|  |  | cbclthoughtprobt | Thought Problems T-score |
| **Memory Performance** | DMS (CANTAB) | dmstotcorz | Total correct responses (Z-score; overall memory accuracy) |
|  |  | dmspercorallz | Percentage of correct responses (Z-score) |
|  |  | dmsmncorlatallz | Mean correct response latency (Z-score; processing speed) |
|  |  | dmsproberrgivcorz | Probability of an error given a correct previous response (Z-score) |
| **Spatial Working Memory** | SWM (CANTAB) | swmtoterrz | Total errors (Z-score; overall working memory performance) |
|  |  | swmstratz | Strategy score (Z-score; efficiency of search strategy) |
|  |  | swmbtwnerrtotz | Between-search errors (Z-score) |
|  |  | swmwinerrtotz | Within-search errors (Z-score) |
| **Adaptive Functioning** | VABS-II | vabsiipadptbehcompstandscore | Adaptive Behavior Composite Standard Score (overall adaptive functioning) |
|  |  | vabsiipcommstandscore | Communication Standard Score |
|  |  | vabsiipdaillivskilstandscore | Daily Living Skills Standard Score |
|  |  | vabsiipsocstandscore | Socialization Standard Score |
|  |  | vabsiipintervscore | Internalizing V-score |
|  |  | vabsiipextervscore | Externalizing V-score |
|  |  | vabsiipmalbehindvscore | Maladaptive Behavior Index V-score |
| **ADHD and Disruptive Behaviors** | DBD | dbdadhdinatt | Number of ADHD inattentive symptoms endorsed (parent/teacher report) |
|  |  | dbdpadhdinatt2 | ADHD Inattention factor score |
|  |  | dbdpadhdimpoa2 | ADHD Hyperactivity/Impulsivity factor score |
|  |  | dbdpodd2 | Oppositional Defiant Disorder factor score |
|  |  | dbdpaverage | Overall behavioral symptom score |

# Supplementary Table 8. Pairwise comparisons from ANCOVA of selected neurobehavioral measures across diagnostic subgroups (AE-FAS, FAS-like, non–FAS-like, and controls)

Pairwise group comparisons were derived from ANCOVA models for each neurobehavioral measure. Models were adjusted for age, sex, and z-scored BMI.

Estimated differences (Estimate) represent adjusted mean differences between groups, with negative values indicating lower scores in Group 1 relative to Group 2. Degrees of freedom (DF) correspond to the residual degrees of freedom from the ANCOVA model.

Reported p-values reflect false discovery rate (FDR)-adjusted p-values across all tests. Comparisons with FDR-adjusted p-values < 0.05 are highlighted.

Group abbreviations are as follows: CON, control; FAS, AE-FAS; FAS-like, facial phenotype consistent with FAS-like subgroup; non–FAS-like, facial phenotype consistent with non–FAS-like subgroup.

Abbreviations for neurobehavioral measures and their corresponding assessment instruments are provided in Supplementary Table 4.

| **Variable** | **DF** | **Group 1** | **Group 2** | **Estimate** | **P** | **Group 1** | **Group 2** | **Estimate** | **P** |
| --- | --- | --- | --- | --- | --- | --- | --- | --- | --- |
| briefpbrit | 207 | CON | FAS | -12.29 | 0.02 | FAS | FAS-like | -0.37 | 1.00 |
| briefpbrit | 207 | CON | FAS-like | -12.67 | 3.00E-05 | FAS | non-FAS-like | 5.37 | 1.00 |
| briefpbrit | 207 | CON | non-FAS-like | -6.92 | 0.25 | FAS-like | non-FAS-like | 5.75 | 0.95 |
| briefpgect | 207 | CON | AS-FAS | -12.47 | 0.01 | FAS | FAS-like | -0.93 | 1.00 |
| briefpgect | 207 | CON | FAS-like | -13.40 | 2.18E-06 | FAS | non-FAS-like | 3.12 | 1.00 |
| briefpgect | 207 | CON | non-FAS-like | -9.34 | 0.01 | FAS-like | non-FAS-like | 4.06 | 1.00 |
| briefpmit | 207 | CON | FAS | -10.91 | 0.02 | FAS | FAS-like | -1.21 | 1.00 |
| briefpmit | 207 | CON | FAS-like | -12.12 | 7.38E-06 | FAS | non-FAS-like | 1.09 | 1.00 |
| briefpmit | 207 | CON | non-FAS-like | -9.82 | 0.01 | FAS-like | non-FAS-like | 2.31 | 1.00 |
| cbcladhdt | 206 | CON | FAS | -9.72 | 1.00E-03 | FAS | FAS-like | 3.64 | 1.00 |
| cbcladhdt | 206 | CON | FAS-like | -6.08 | 1.25E-03 | FAS | non-FAS-like | 4.20 | 1.00 |
| cbcladhdt | 206 | CON | non-FAS-like | -5.52 | 0.03 | FAS-like | non-FAS-like | 0.56 | 1.00 |
| cbclaggbehavt | 207 | CON | FAS | -12.20 | 2.52E-04 | FAS | FAS-like | 4.12 | 1.00 |
| cbclaggbehavt | 207 | CON | FAS-like | -8.09 | 2.52E-04 | FAS | non-FAS-like | 6.71 | 0.39 |
| cbclaggbehavt | 207 | CON | non-FAS-like | -5.49 | 0.14 | FAS-like | non-FAS-like | 2.59 | 1.00 |
| cbclanxdeprt | 207 | CON | FAS | -4.06 | 0.52 | FAS | FAS-like | -1.54 | 1.00 |
| cbclanxdeprt | 207 | CON | FAS-like | -5.60 | 1.19E-03 | FAS | non-FAS-like | 2.27 | 1.00 |
| cbclanxdeprt | 207 | CON | non-FAS-like | -1.79 | 1.00 | FAS-like | non-FAS-like | 3.81 | 0.52 |
| cbclattprobt | 207 | CON | FAS | -11.94 | 2.61E-04 | FAS | FAS-like | 4.97 | 0.70 |
| cbclattprobt | 207 | CON | FAS-like | -6.96 | 1.22E-03 | FAS | non-FAS-like | 4.22 | 1.00 |
| cbclattprobt | 207 | CON | non-FAS-like | -7.72 | 2.51E-03 | FAS-like | non-FAS-like | -0.75 | 1.00 |
| cbclexternalprobt | 207 | CON | FAS | -11.79 | 0.01 | FAS | FAS-like | 2.24 | 1.00 |
| cbclexternalprobt | 207 | CON | FAS-like | -9.55 | 4.66E-04 | FAS | non-FAS-like | 4.52 | 1.00 |
| cbclexternalprobt | 207 | CON | non-FAS-like | -7.27 | 0.06 | FAS-like | non-FAS-like | 2.28 | 1.00 |
| cbclinternalprobt | 207 | CON | FAS | -8.15 | 0.10 | FAS | FAS-like | 0.42 | 1.00 |
| cbclinternalprobt | 207 | CON | FAS-like | -7.72 | 2.94E-03 | FAS | non-FAS-like | 2.78 | 1.00 |
| cbclinternalprobt | 207 | CON | non-FAS-like | -5.37 | 0.24 | FAS-like | non-FAS-like | 2.36 | 1.00 |
| cbclsocprobt | 186 | CON | FAS | -9.89 | 4.39E-04 | FAS | FAS-like | 2.50 | 1.00 |
| cbclsocprobt | 186 | CON | FAS-like | -7.39 | 9.40E-05 | FAS | non-FAS-like | 7.92 | 0.04 |
| cbclsocprobt | 186 | CON | non-FAS-like | -1.98 | 1.00E+00 | FAS-like | non-FAS-like | 5.42 | 0.09 |
| cbclsomcomplt | 207 | CON | FAS | -4.80 | 0.53 | FAS | FAS-like | -0.94 | 1.00 |
| cbclsomcomplt | 207 | CON | FAS-like | -5.74 | 3.62E-03 | FAS | non-FAS-like | 1.30 | 1.00 |
| cbclsomcomplt | 207 | CON | non-FAS-like | -3.50 | 0.53 | FAS-like | non-FAS-like | 2.24 | 1.00 |
| cbclthoughtprobt | 186 | CON | FAS | -9.25 | 4.13E-03 | FAS | FAS-like | 1.95 | 1.00 |
| cbclthoughtprobt | 186 | CON | FAS-like | -7.30 | 5.39E-04 | FAS | non-FAS-like | 6.53 | 0.31 |
| cbclthoughtprobt | 186 | CON | non-FAS-like | -2.72 | 1.00 | FAS-like | non-FAS-like | 4.59 | 0.39 |
| cbcltotprobt | 207 | CON | FAS | -13.57 | 5.61E-04 | FAS | FAS-like | 3.62 | 1.00 |
| cbcltotprobt | 207 | CON | FAS-like | -9.95 | 1.50E-04 | FAS | non-FAS-like | 5.70 | 1.00 |
| cbcltotprobt | 207 | CON | non-FAS-like | -7.87 | 0.02 | FAS-like | non-FAS-like | 2.08 | 1.00 |
| dbdadhdinatt | 237 | CON | FAS | -1.35 | 0.66 | FAS | FAS-like | 1.29 | 0.84 |
| dbdadhdinatt | 237 | CON | FAS-like | -0.06 | 1.00 | FAS | non-FAS-like | -0.92 | 1.00 |
| dbdadhdinatt | 237 | CON | non-FAS-like | -2.28 | 0.01 | FAS-like | non-FAS-like | -2.21 | 0.03 |
| dbdpadhdimpoa2 | 237 | CON | FAS | 0.10 | 1.00 | FAS | FAS-like | -0.20 | 1.00 |
| dbdpadhdimpoa2 | 237 | CON | FAS-like | -0.09 | 1.00 | FAS | non-FAS-like | -0.38 | 1.00 |
| dbdpadhdimpoa2 | 237 | CON | non-FAS-like | -0.28 | 1.00 | FAS-like | non-FAS-like | -0.18 | 1.00 |
| dbdpadhdinatt2 | 237 | CON | FAS | 0.05 | 1.00 | FAS | FAS-like | -0.04 | 1.00 |
| dbdpadhdinatt2 | 237 | CON | FAS-like | 0.01 | 1.00 | FAS | non-FAS-like | -0.41 | 0.76 |
| dbdpadhdinatt2 | 237 | CON | non-FAS-like | -0.36 | 0.64 | FAS-like | non-FAS-like | -0.38 | 0.64 |
| dbdpaverage | 237 | CON | FAS | 0.09 | 1.00 | FAS | FAS-like | -0.10 | 1.00 |
| dbdpaverage | 237 | CON | FAS-like | -0.01 | 1.00 | FAS | non-FAS-like | -0.35 | 0.28 |
| dbdpaverage | 237 | CON | non-FAS-like | -0.26 | 0.28 | FAS-like | non-FAS-like | -0.25 | 0.36 |
| dbdpodd2 | 237 | CON | FAS | 0.14 | 1.00 | FAS | FAS-like | -0.08 | 1.00 |
| dbdpodd2 | 237 | CON | FAS-like | 0.06 | 1.00 | FAS | non-FAS-like | -0.53 | 0.15 |
| dbdpodd2 | 237 | CON | non-FAS-like | -0.38 | 0.15 | FAS-like | non-FAS-like | -0.44 | 0.15 |
| dmsmncorlatallz | 205 | CON | FAS | -0.25 | 1.00 | FAS | FAS-like | 0.28 | 1.00 |
| dmsmncorlatallz | 205 | CON | FAS-like | 0.03 | 1.00 | FAS | non-FAS-like | 0.68 | 0.97 |
| dmsmncorlatallz | 205 | CON | non-FAS-like | 0.43 | 0.97 | FAS-like | non-FAS-like | 0.40 | 1.00 |
| dmspercorallz | 205 | CON | FAS | 0.55 | 0.22 | FAS | FAS-like | -0.15 | 1.00 |
| dmspercorallz | 205 | CON | FAS-like | 0.40 | 0.22 | FAS | non-FAS-like | -0.66 | 0.22 |
| dmspercorallz | 205 | CON | non-FAS-like | -0.11 | 1.00 | FAS-like | non-FAS-like | -0.52 | 0.22 |
| dmsproberrgivcorz | 205 | CON | FAS | 0.56 | 0.27 | FAS | FAS-like | -0.09 | 1.00 |
| dmsproberrgivcorz | 205 | CON | FAS-like | 0.47 | 0.11 | FAS | non-FAS-like | -0.59 | 0.34 |
| dmsproberrgivcorz | 205 | CON | non-FAS-like | -0.04 | 1.00 | FAS-like | non-FAS-like | -0.51 | 0.27 |
| dmstotcorz | 205 | CON | FAS | 0.55 | 0.28 | FAS | FAS-like | -0.20 | 1.00 |
| dmstotcorz | 205 | CON | FAS-like | 0.35 | 0.28 | FAS | non-FAS-like | -0.65 | 0.28 |
| dmstotcorz | 205 | CON | non-FAS-like | -0.10 | 1.00 | FAS-like | non-FAS-like | -0.45 | 0.37 |
| swmbtwnerrtotz | 205 | CON | FAS | 0.70 | 0.05 | FAS | FAS-like | -0.50 | 0.42 |
| swmbtwnerrtotz | 205 | CON | FAS-like | 0.21 | 1.00 | FAS | non-FAS-like | -0.58 | 0.42 |
| swmbtwnerrtotz | 205 | CON | non-FAS-like | 0.12 | 1.00 | FAS-like | non-FAS-like | -0.09 | 1.00 |
| swmstratz | 235 | CON | FAS | 0.06 | 1.00 | FAS | FAS-like | 0.24 | 1.00 |
| swmstratz | 235 | CON | FAS-like | 0.30 | 1.00 | FAS | non-FAS-like | 0.20 | 1.00 |
| swmstratz | 235 | CON | non-FAS-like | 0.26 | 1.00 | FAS-like | non-FAS-like | -0.04 | 1.00 |
| swmtoterrz | 235 | CON | FAS | 0.01 | 1.00 | FAS | FAS-like | 0.68 | 0.06 |
| swmtoterrz | 235 | CON | FAS-like | 0.69 | 4.48E-04 | FAS | non-FAS-like | 0.73 | 0.07 |
| swmtoterrz | 235 | CON | non-FAS-like | 0.74 | 2.54E-03 | FAS-like | non-FAS-like | 0.05 | 1.00 |
| swmwinerrtotz | 235 | CON | FAS | -0.15 | 1.00 | FAS | FAS-like | 0.40 | 0.25 |
| swmwinerrtotz | 235 | CON | FAS-like | 0.25 | 0.25 | FAS | non-FAS-like | 0.57 | 0.10 |
| swmwinerrtotz | 235 | CON | non-FAS-like | 0.41 | 0.10 | FAS-like | non-FAS-like | 0.16 | 1.00 |
| Vabsiipadptbeh-  compstandscore | 205 | CON | FAS | -5.81 | 1.00 | FAS | FAS-like | 7.58 | 0.99 |
| Vabsiipadptbeh-  compstandscore | 205 | CON | FAS-like | 1.76 | 1.00 | FAS | non-FAS-like | 15.55 | 0.13 |
| V  absiipadptbeh-  compstandscore | 205 | CON | non-FAS-like | 9.74 | 0.20 | FAS-like | non-FAS-like | 7.97 | 0.99 |
| Vabsiipcommstand-  score | 233 | CON | FAS | -2.82 | 1.00 | FAS | FAS-like | 2.29 | 1.00 |
| Vabsiipcommstand-  score | 233 | CON | FAS-like | -0.53 | 1.00 | FAS | non-FAS-like | 7.89 | 1.00 |
| Vabsiipcommstand-  score | 233 | CON | non-FAS-like | 5.07 | 1.00 | FAS-like | non-FAS-like | 5.60 | 1.00 |
| Vabsiipdaillivskil-  standscore | 237 | CON | FAS | -9.65 | 0.60 | FAS | FAS-like | 12.49 | 0.42 |
| Vabsiipdaillivskil-  standscore | 237 | CON | FAS-like | 2.85 | 1.00 | FAS | non-FAS-like | 10.92 | 0.62 |
| Vabsiipdaillivskil-  standscore | 237 | CON | non-FAS-like | 1.28 | 1.00 | FAS-like | non-FAS-like | -1.57 | 1.00 |
| vabsiipextervscore | 234 | CON | FAS | 0.20 | 1.00 | FAS | FAS-like | 0.14 | 1.00 |
| vabsiipextervscore | 234 | CON | FAS-like | 0.35 | 1.00 | FAS | non-FAS-like | -1.06 | 1.00 |
| vabsiipextervscore | 234 | CON | non-FAS-like | -0.86 | 1.00 | FAS-like | non-FAS-like | -1.21 | 1.00 |
| vabsiipintervscore | 233 | CON | FAS | 0.16 | 1.00 | FAS | FAS-like | 0.33 | 1.00 |
| vabsiipintervscore | 233 | CON | FAS-like | 0.49 | 1.00 | FAS | non-FAS-like | -1.66 | 0.68 |
| vabsiipintervscore | 233 | CON | non-FAS-like | -1.49 | 0.25 | FAS-like | non-FAS-like | -1.98 | 0.17 |
| Vabsiipmalbehindv-  score | 233 | CON | FAS | 0.43 | 1.00 | FAS | FAS-like | 0.13 | 1.00 |
| Vabsiipmalbehindv-  score | 233 | CON | FAS-like | 0.56 | 1.00 | FAS | non-FAS-like | -1.59 | 0.78 |
| Vabsiipmalbehindv-  score | 233 | CON | non-FAS-like | -1.16 | 0.78 | FAS-like | non-FAS-like | -1.72 | 0.47 |
| vabsiipsocstandscore | 234 | CON | FAS | -4.53 | 1.00 | FAS | FAS-like | 3.44 | 1.00 |
| vabsiipsocstandscore | 234 | CON | FAS-like | -1.09 | 1.00 | FAS | non-FAS-like | 8.56 | 1.00 |
| vabsiipsocstandscore | 234 | CON | non-FAS-like | 4.03 | 1.00 | FAS-like | non-FAS-like | 5.12 | 1.00 |

# Supplementary Figure S1. Boxplot distributions of age, height, weight, body mass index (BMI) z score across study groups

# Supplementary Figure S2. Boxplot distributions of size-normalized (scaled) philtrum length, nasal protrusion and mandibular arc length across study groups

# Supplementary Figure S3. Regional facial signatures derived from the size-normalized (scaled) dense surface models for alcohol-exposed subgroups relative to controls

For each facial region, (A) eyes, (B) malar region, (C) nose, (D) lip–philtrum, (E) zygomatic arch, and (F) profile, the upper row shows the mean facial signature for the AE-FAS group, and the lower row shows the mean facial signature for the AE group.

Within each panel, the first column represents the overall regional mean signature, while the remaining columns show displacements along the lateral (X), vertical (Y), and depth (Z) axes, respectively.

Color-coded heatmaps (red–green–blue) represent contraction, minimal change, and expansion along the surface normal relative to age-matched controls, at the indicated significance thresholds.
